# Dual mechanism of the OXA-23 carbapenemase inhibition by the carbapenem NA-1-157

**DOI:** 10.1128/aac.00918-25

**Published:** 2025-08-20

**Authors:** Marta Toth, Nichole K. Stewart, Pojun Quan, Md Mahbub Kabir Khan, Jonathan Cox, John D. Buynak, Clyde A. Smith, Sergei B. Vakulenko

**Affiliations:** 1 Department of Chemistry and Biochemistry, University of Notre Dame 6111, Notre Dame, Indiana, USA; 2 Department of Chemistry, Southern Methodist University 427637, Dallas, Texas, USA; 3 Stanford Synchrotron Radiation Lightsource, Stanford University 6429, Menlo Park, California, USA; 4 Department of Chemistry, Stanford University 198873, Stanford, California, USA; University of Fribourg, Fribourg, Switzerland

**Keywords:** carbapenemase, mechanism of inhibition, inhibitor, antibiotic resistance, *Acinetobacter baumannii*

## Abstract

Carbapenem-resistant *Acinetobacter baumannii* continues to be a leading cause of life-threatening infections that result in high mortality rates. The major cause of carbapenem resistance in this pathogen is the production of class D carbapenemases, enzymes that inactivate the last resort carbapenem antibiotics, thus significantly diminishing the available therapeutic options. In this study, we evaluated the interaction of OXA-23, the most widely disseminated class D carbapenemase in *A. baumannii* clinical isolates, with the atypically modified carbapenem, NA-1-157. The MICs of this compound against strains producing OXA-23 were reduced from highly resistant levels observed for the commercial carbapenems meropenem and imipenem (16–128 µg/mL) to sensitive or intermediate levels (2–4 µg/mL). Kinetic studies showed that NA-1-157 inhibits the enzyme due to a significant decrease (>2,000-fold) in the deacylation rate relative to its closest structural analog, meropenem. Structural studies and molecular dynamics simulations demonstrated that inhibition is caused by both the inability of a water molecule to get close enough to the scissile bond to perform deacylation and by partial decarboxylation of the catalytic lysine residue upon formation of the acyl-enzyme intermediate.

## INTRODUCTION

Antibiotic resistance in bacteria is one of the leading causes of death globally (1). Of particular concern is the spread of multidrug-resistant (MDR) microorganisms for which therapeutic options are very limited. Among them, carbapenem-resistant *Acinetobacter baumannii*, a Gram-negative opportunistic bacterium, is recognized by the CDC as a pathogen that presents the highest level of threat to human health (2). Prior to the spread of such bacteria, carbapenem antibiotics were the drugs of choice for treatment of *A. baumannii* infections. Over time, MDR *A. baumannii* has spread in clinics worldwide (up to 80% on average) (3), causing difficult-to-treat hospital-acquired infections, such as ventilator-associated pneumonia, bacteremia, urinary tract and wound infections, and endocarditis (4). These infections often result in high mortality rates, reaching 85% or even higher (5–8). Because of the lack of adequate treatment options for infections caused by MDR *A. baumannii*, there is a critical need to develop effective therapies that are active against this pathogen.

Resistance to carbapenems in *A. baumannii* can be caused by several mechanisms (alteration of the β-lactam target, the penicillin binding proteins (PBPs), modification or loss of porins, and upregulated efflux), but production of β-lactamases, enzymes that hydrolyze the β-lactam antibiotics, is by far the prevailing cause (9). β-Lactamases are categorized into four molecular classes, with class A, C, and D enzymes utilizing an active-site serine for catalysis, while class B enzymes are zinc-dependent metallo-enzymes. β-Lactamases, capable of inactivating carbapenem antibiotics (carbapenemases), are found among enzymes of classes A, B, and D, with those from class D being the most prevalent cause of carbapenem resistance in *A. baumannii*. The major difference in the mechanism of β-lactam antibiotic hydrolysis by the active-site serine enzymes is the use of different amino acid residues for deacylation of the acyl-enzyme intermediate (glutamate for class A, tyrosine for class C, and a carboxylated lysine for class D) (10, 11). In *A. baumannii*, the carbapenem-hydrolyzing class D β-lactamases or CHDLs fall into seven main groups (the acquired OXA-23-like, OXA-24/40-like, OXA-58-like, OXA-134-like, and OXA-143-like enzymes, and the intrinsic OXA-51-like and OXA-213-like β-lactamases) based on amino acid sequence identity (Table S1) (12). Of these, the carbapenemase OXA-23 is by far the most common (~70%) in *A. baumannii* clinical isolates (13).

**Fig 1:**
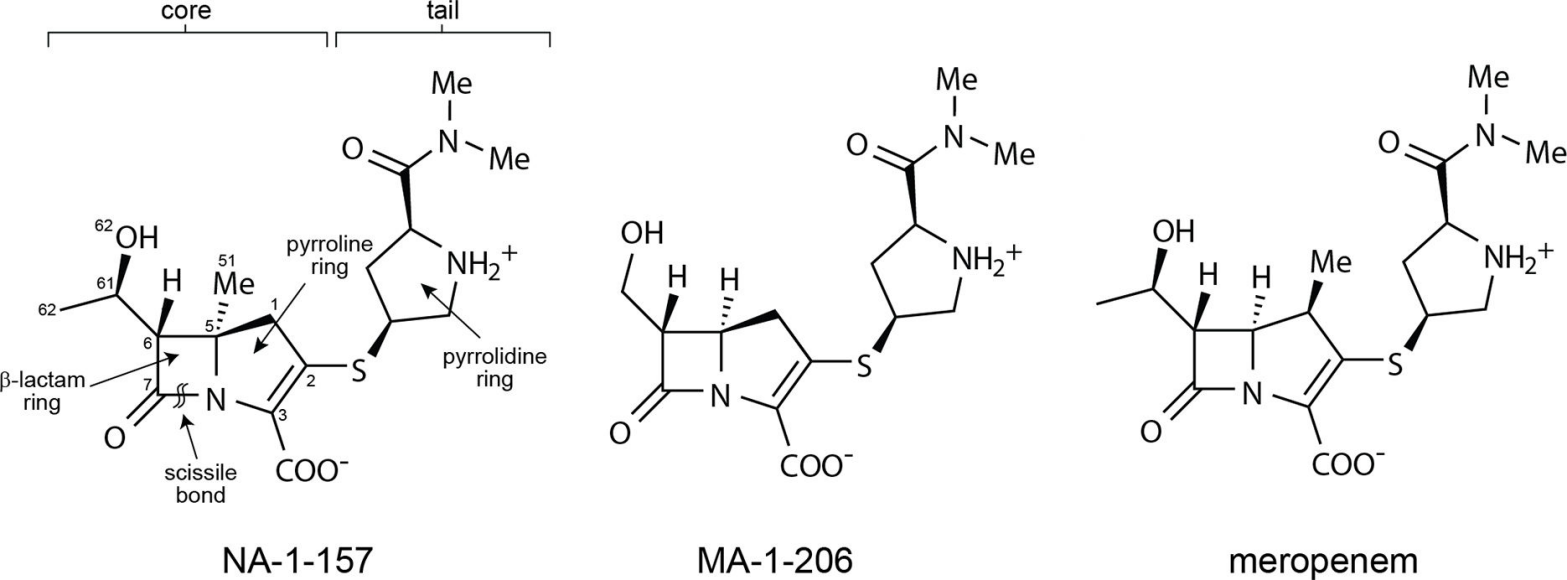
Chemical structures of the atypical carbapenems NA-1-157 and MA-1-206, and the commercial carbapenem meropenem, a substrate of CHDLs.

Due to the wide dissemination of CHDLs, new antibiotics resistant to degradation by these enzymes, as well as inhibitors targeting them, are urgently needed. Carbapenems are an attractive class of antibiotics for further development, as they are well-studied drugs that exhibit low toxicity and good pharmacokinetic and pharmacodynamic properties (14, 15). In an effort to increase the efficacy of these drugs, we have undertaken the task of modifying the canonical carbapenem scaffold. Previously, we showed that substitution of the 6α-hydroxyethyl (6α-HE) group with a 6α-hydroxymethyl in the carbapenem MA-1-206 (Fig. 1) transformed the compound from a substrate to a reversible inhibitor of the CHDL OXA-23 (16). We also demonstrated that the presence of the C5α-methyl group in NA-1-157 severely impacts its turnover by OXA-58 (17) and OXA-48 (18), the latter being the most common class D carbapenemase found in Enterobacterales. Additionally, we found that this compound is a potent inhibitor of GES-5 (19), a class A carbapenemase, thus demonstrating its activity against β-lactamases from different molecular classes. In this study, we investigated the interaction of NA-1-157 with the major *A. baumannii* CHDL, OXA-23.

## RESULTS AND DISCUSSION

### Antibiotic susceptibility testing

Previously, we demonstrated that NA-1-157 has good microbiological activity against isogenic *A. baumannii* strains producing various CHDLs from an *Escherichia coli–A. baumannii* shuttle vector (17). For strains expressing the majority of the CHDLs (OXA-23, OXA-24/40, OXA-51, and OXA-58), the MICs of the compound were in the sensitive to intermediate range (1–4 µg/mL), while for that producing OXA-143, the MIC was 8 µg/mL. We also assessed the compound’s efficacy against 40 genotypically characterized MDR CHDL-producing *A. baumannii* clinical isolates from the CDC & FDA Antibiotic Resistance (AR) Isolate Bank (20). We observed a similar trend, where the MICs of NA-1-157 against bacteria producing OXA-23, OXA-24/40, and OXA-58 were in the sensitive to intermediate range (2–4 µg/mL), while those for OXA-72 producers were 4–8 µg/mL (Table 1). Importantly, none of the isolates (*n* = 23) that expressed the most clinically relevant CHDL OXA-23 were resistant to NA-1-157, though they were highly resistant to the commercial carbapenems meropenem and imipenem, with MICs of 32–128 and 16–64 µg/mL, respectively (Table 1).

**TABLE 1:** MICs (µg/mL) of β-lactams against MDR *A. baumannii* clinical isolates

Enzyme*^a^*	MIC (µg/mL)			Number of strains
	NA-1-157	Meropenem*^b^*	Imipenem*^b^*	
OXA-23	2–4	32–128	16–64	22
OXA-23, OXA-24/40	2	128	64	1
OXA-24/40	2–4	128–256	64–128	8
OXA-58	2	8	8	2
OXA-72	4–8	256–512	64–128	7

^*a*^ All strains encode OXA-51 (such as OXA-65, -66, -69, -82, -94, -100, -203, and -223) and ADC derivatives. Eighteen strains encode TEM-type, and one encodes PER-7 β-lactamases.

^*b*^ These data were previously published (16).

### Evaluation of kinetics

Under steady-state conditions, the total change in absorbance for the reaction between OXA-23 and NA-1-157 was less than expected for acylation of all the enzyme (Fig. S1A), suggesting that the reaction was biphasic and part of it (a faster phase) completed during the manual mixing time, as was observed with the OXA-58 and GES-5 carbapenemases (17, 19). Using a stopped-flow apparatus, we were able to observe this fast phase of acylation, which comprised only ~10% of the total reaction. The progress curve at later time points also showed that no or only very slow deacylation occurred (Fig. S1A), indicating that for clinical purposes, NA-1-157 is an inhibitor of the enzyme.

Next, the acylation rate constants of the biphasic reaction were evaluated using single turnover conditions. For the fast phase, saturation could not be reached with the highest possible enzyme concentration (250 µM); under this condition, the acylation rate constant *k*_2 fast_ (Scheme 1; Fig. S2) was determined to be >26 ± 1 s^−1^ (Fig. S1B; Table 2). For the slow phase, comprising the majority (~90%) of the reaction, the acylation rate constant *k*_2 slow_ (Scheme 1) was (7.1 ± 0.4) × 10^−3^ s^−1^ (Fig. S1C; Table 2), which is more than 3,600-fold slower than *k*_2 fast_. This rate is ~8-fold slower than that of meropenem. These results show that both phases of acylation of OXA-23 by NA-1-157 are slowed by the presence of the C5α-methyl group of the compound when compared to the commercial carbapenem meropenem.

**TABLE 2:** Kinetic parameters for the interaction of carbapenemases with NA-1-157 or meropenem

Parameter	Enzyme				
	OXA-23 with Meropenem*^a^*	OXA-23 with NA-1-157	OXA-58 with NA-1-157*^a^*	OXA-48 with NA-1-157*^a^*	GES-5 with NA-1-157*^a^*
*k*_2_ fast (s^−1^)*^b^*	≥330 (30)	>26 ± 1 (10)	1.0 ± 0.3 (25)	UD^*c*^	99 ± 31 (46)
*k*_2_ slow (s^−1^)*^b^*	(5.6 ± 0.1) × 10^−2^ (70)	(7.1 ± 0.4) × 10^−3^ (90)	(8.6 ± 0.6) × 10^−4^ (75)	(8.4 ± 0.6) × 10^−3^ (≥95)	(2.6 ± 0.1) × 10^−4^ (54)
*k*_3_ (s^−1^)	0.12 ± 0.01	(5.5 ± 0.3) × 10^−5^	(9.1 ± 0.3) × 10^−5^	0.15 ± 0.01	(2.4 ± 0.3) × 10^−7^
*k*_NA-1-157_ (s^−1^)	NA*^d^*	(6.8 ± 0.3) × 10^−3^	0.55 ± 0.05	NA	4.1 ± 0.8
*K*_I_ (µM)	NA	0.65 ± 0.07	1.9 ± 0.3	NA	14 ± 3
*k*_NA-1-157_/*K*_I_ (M^−1^s^−1^)	NA	(1.1 ± 0.1) × 10^4^	(2.9 ± 0.5) × 10^5^	NA	(2.9 ± 0.9) × 10^5^
*K*_*s*_ or *K*_*i*_ (µM)	0.06 ± 0.01	0.59 ± 0.02	–	0.22 ± 0.01	–
Rate-limiting step	Slow acylation	Deacylation	Deacylation	Slow acylation	Deacylation
Substrate or inhibitor	Substrate	Inhibitor	Inhibitor	Poor substrate	Inhibitor

^*a*^ Data were reported in earlier publications (16–19).

^*b*^ The second number in parenthesis for *k*_2 fast_ and *k*_2 slow_ gives the percentage of the fast and slow phases of the acylation reaction, respectively.

^*c*^ UD, unable to determine.

^*d*^ NA, not applicable.

To determine whether any deacylation of the acyl-enzyme complex occurs, we performed a jump dilution experiment (21) using a discontinuous assay. This showed that the activity of the enzyme was restored only very slowly (Fig. S1D), with a *k*_3_ rate constant (Scheme 1) of (5.5 ± 0.3) × 10^−5^ s^−1^ (Table 2). This deacylation rate of NA-1-157 would result in a residence time of 303 ± 17 min, which is ~2180-fold longer than that of meropenem. This residence time is also >10-fold longer than the *A. baumannii* CIP 70.10 doubling rate of 22 ± 0.4 min (22), further showing that, for clinical purposes, NA-1-157 is an inhibitor of OXA-23. These results reveal that the rates of both acylation and deacylation of NA-1-157 are impaired when compared to the other two commercial carbapenems; however, inhibition of OXA-23 by the compound is caused by severely compromised deacylation.

Plotting the *k*_inter_ values obtained from a competition experiment with nitrocefin versus concentration of NA-1-157 (Fig. S1E) allowed us to determine the parameters *k*_NA-1-157_, which is the maximum rate of inactivation of OXA-23 resulting from formation of both an irreversible and reversible species (19) (formation of the latter is described in the next section [19]), and *K*_I_ representing the concentration of NA-1-157 required to reach one-half of *k*_NA-1-157_ (analogous to *K*_*m*_ for substrates). The *k*_NA-1-157_ value ((6.8 ± 0.3) × 10^−3^ s^−1^ (Table 2)) shows that the half-life of inactivation of OXA-23 is 102 ± 5 s. This parameter is nearly identical to *k*_2 slow_, which suggests that the slower phase of acylation is the major contributor to the observed rate of inactivation. The parameter *K*_I_ was submicromolar (0.65 ± 0.07 µM), indicating that OXA-23 has good apparent affinity for the compound. The second-order rate constant *k*_inact_/*K*_I_ was calculated to be (1.1 ± 0.1) × 10^4^ M^−1^s^−1^, showing that the inhibition efficiency is moderate. The *K*_*i*_ value (0.59 ± 0.02 µM, Fig. S1F; Table 2), representing the binding affinity in the noncovalent Michaelis complex, was ~10-fold weaker than the equivalent parameter (*K*_*s*_) for meropenem (Table 2). Despite this decrease in affinity, the enzyme would be fully saturated at the MIC of NA-1-157 (2–4 µg/mL or 5–10 µM). Taken all together, these data demonstrate that the presence of the C5α-methyl group in NA-1-157 dramatically increases the stability of this carbapenem against hydrolysis by OXA-23, the most commonly found carbapenemase in *A. baumannii*.

Previously, we described the interaction of NA-1-157 with three other carbapenemases: GES-5 from class A, and OXA-48 and OXA-58 from class D β-lactamases (17–19). Among them, two phases of acylation (fast and slow) were detected for all the enzymes except OXA-48, where either the reaction is monophasic or the fast phase is below the detection level (Table 2). The ratio of the fast and slow phases varied significantly, from 46:54 (for GES-5) to 10:90 (for OXA-23). These studies also demonstrated that while OXA-48 has the fastest *k*_2 slow_ rate, it is the only one of the four enzymes whose rate-limiting step is acylation. OXA-48 also has the fastest deacylation rate (*k*_3_), and as a result, NA-1-157 acts as a poor substrate. The other three enzymes have much slower deacylation rates, with NA-1-157 residence times ranging from 3 hours to 48 days, which shows that for clinical purposes, the compound is a potent inhibitor. Comparison of the inhibition parameters of the enzymes shows that while OXA-23 has the highest apparent affinity (*K*_I_), its rate of inactivation (*k*_NA-1-157_) is significantly slower, resulting in the lowest inhibition efficiency (*k*_NA-1-157_/*K*_I_) when compared to OXA-58 and GES-5. This may contribute to the observed higher MIC of NA-1-157 against *A. baumannii* expressing OXA-23 in comparison to MICs against strains producing OXA-58 (Table 1) or GES-5 (17, 19).

### Electrospray ionization-liquid chromatography/mass spectrometry (ESI-LC/MS)

Previously, upon reaction of NA-1-157 with the OXA-58 and GES-5 carbapenemases, we observed by ESI-LC/MS formation of a pre-acylation covalent species and proposed that it may be represented by a reversible tetrahedral intermediate (Fig. S2) (17, 19). We performed the same experiment with OXA-23, which showed that after only 5 s (the minimal time required for mixing and quenching), nearly all the enzyme was in a covalent complex with the inhibitor (Fig. S3A). According to our present kinetic experiments, only a small amount (<20%) of acyl-enzyme can form during this short time period. This indicates that the remaining 80% of the covalent complex observed by ESI-LC/MS is represented by a pre-acylation intermediate(s). Our subsequent competition experiment using another carbapenem, PQ-1-219, showed that this intermediate(s) is reversible (Fig. S3B). This experiment also demonstrated that incubation of OXA-23 with NA-1-157 for longer time periods (30, 90, and 300 s) resulted in an increase of the covalent OXA-23-NA-1-157 acyl-enzyme intermediate to 29%, 53%, and 82%, respectively, which is in full agreement with our kinetic data.

The phenomenon of formation of a reversible covalent species with NA-1-157 has been observed by us with three different β-lactamases: GES-5 (class A) (19), OXA-58 (class D) (17), and OXA-23 (class D) (this study). These species, whose detection was possible due to very slow acylation and deacylation rates, provide insights into the nature of the observed slow and fast phases of acylation. The data show that for all three enzymes, the percentage of reversible species detected by MS at the earliest time point is the same as the percentage of enzyme acylated during the slow phase of the reaction in our kinetic experiments. Also, the rate of conversion of the reversible covalent pre-acylation intermediate(s) into the acyl-enzyme (data not shown) is very similar to the rate constant *k*_2 slow_. Based on these results, we propose that the slow phase of acylation is determined by the sluggish rate of conversion of the reversible covalent pre-acylation intermediate(s) into the acyl-enzyme. This implies that during the fast phase of acylation, the reaction must proceed through a different intermediate(s), which is converted to the acyl-enzyme much more efficiently. The existence of fast and slow phases of acylation was first demonstrated for the interaction of the class A GES β-lactamases with commercial carbapenems (23, 24), and subsequently reported for another class A enzyme, KPC-2 (25). Later, it was also observed for the class D enzyme OXA-23 (16), and recently for the class C enzyme ADC-1 (26), thus expanding this phenomenon to all classes of serine β-lactamases. It is likely that in all of these cases, fast and slow acylation also proceed through different pre-acylation intermediates.

### Crystal structures of apo-OXA-23 and its meropenem complex

**Fig 2:**
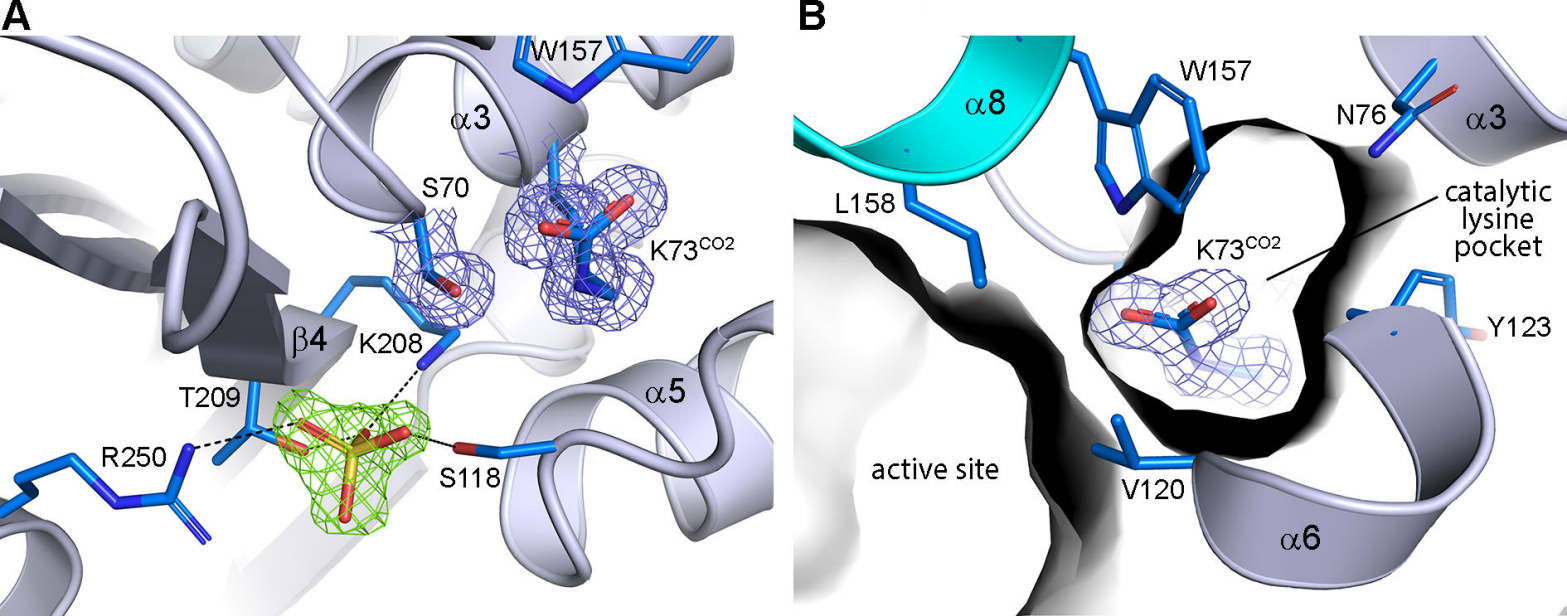
Apo-OXA-23 structure. (**A**) Active site showing the bound sulfate (yellow/red sticks) in *F_o_–F_c_* density (green mesh, 3.5 σ). 2*F_o_–F_c_* density (blue mesh, 1.2 σ) is shown for the catalytic Ser70 and the carboxylated lysine, Lys73^CO2^. (**B**) Cutaway view into the internal CLP, which is separated from the active site by the two hydrophobic residues, Val120 and Leu158, which form a hydrophobic cap.

In the apo-OXA-23 structure (Fig. S4), the catalytic lysine residue (Lys73^CO2^ in the standard class D numbering [12, 27]) is fully carboxylated (Fig. 2A) and sequestered in an internal pocket formed by the side chains of Asn76, Tyr123, and Trp157, as well as the side of helix α3 (Fig. 2B). The catalytic lysine pocket (CLP) is separated from the active site and the external milieu by a hydrophobic cap (Fig. S5A), formed by the side chains of Val120 and Leu158 (Fig. 2B; Fig. S5B) (28, 29). A sulfate ion is observed in the active site, interacting with the Arg250 side chain.

In the OXA-23–meropenem acyl-enzyme complex, meropenem is covalently attached to the Ser70 side chain (Fig. S6A) and in the Δ^2^ tautomeric conformation (Fig. S6B), with its tail (the pyrrolidine ring and the dimethylcarbamoyl group) conformationally disordered. The core of the substrate is bound in the canonical conformation, with the O7 carbonyl oxygen positioned in the oxyanion hole formed by the main chain nitrogen atoms of Ser70 and Trp211, and the C3 carboxylate hydrogen bonded to Thr209 and Arg250. The 6α-HE group of meropenem is inward-facing with respect to the catalytic lysine (17, 19), with its O62 atom hydrogen bonded to a fully occupied Lys73^CO2^ (Fig. S6B). The C62 atom forces Val120 into the less-favored open conformation, while the Leu158 side chain, the second residue forming the hydrophobic cap over the CLP, remains in the closed conformation (Fig. S6C).

### Time-resolved binding of NA-1-157 to OXA-23

**Fig 3:**
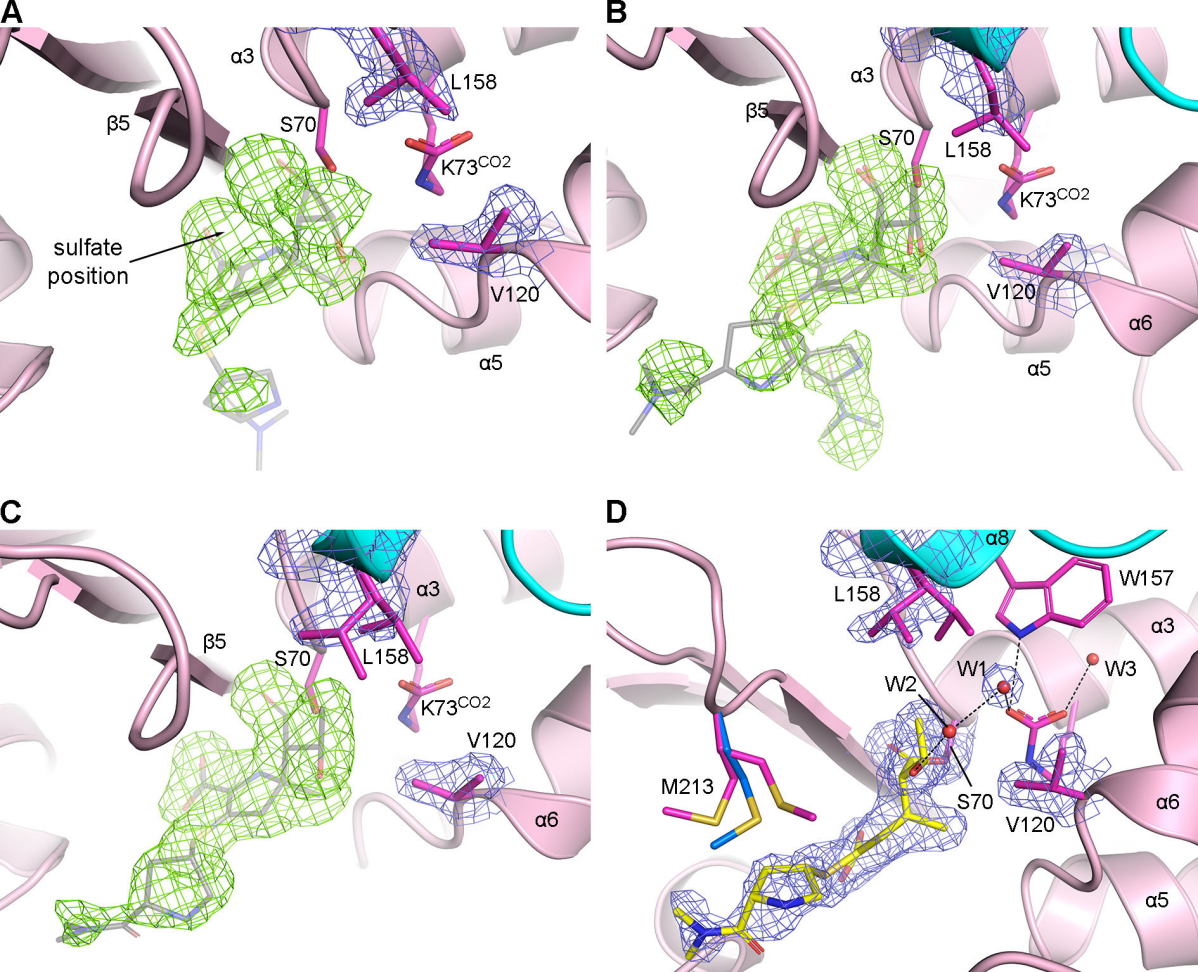
Time-resolved OXA-23–NA-1-157 complexes. (**A**) The *t* = 3 min time point. (**B**) The *t* = 4 min time point. (**C**) The *t* = 6 min time point. The 2*F_o_–F_c_* density for Val120 and Leu158 (blue mesh, 1.0 σ), polder density (green mesh, 3.5 σ), and the final positions of NA-1-157 (semi-transparent gray sticks) at each time point are shown in **A**, **B**, and **C**. (**D**) Final 2*F_o_–F_c_* density (blue mesh, 1.0 σ) for the Ser70, Val120, and Leu158 side chains in the *t* = 6 min OXA-23–NA-1-157 complex. NA-1-157 is shown as yellow sticks. Two rotamers of Met213 are shown for the inhibitor complex (magenta sticks), and the rotamer in apo-OXA-23 is shown as blue sticks for comparison.

Four time points (3, 4, 6, and 10 min) were used to analyze the binding of NA-1-157 to OXA-23. At *t* = 3 min, the sulfate anion is still visible in the active site, with additional *F_o_–F_c_* difference density peaks attributed to either solvent or low occupancy of the inhibitor. A polder density map (30), which excludes the solvent contribution in the active site, clearly shows a large piece of density roughly matching the shape of the core of NA-1-157 (refined at ~50% occupancy), in conjunction with a partially occupied sulfate molecule (Fig. 3A). Although the polder density for the tail group beyond the exocyclic sulfur is very weak, the position of the tail could be discerned.

At *t* = 4 min, the presence of the acyl-enzyme intermediate is much clearer (Fig. 3B). Lobes of density for the C3 carboxylate and the 6α-HE groups are evident, and the pyrroline ring is in the Δ^2^ tautomerization state. Density beyond the exocyclic sulfur atom suggests two alternate positions of the tail group at approximately equal occupancies. The occupancy of the Lys73^CO2^ carboxylate refined to <100%. This was subsequently refined to give occupancies of 70% and 30% for carboxylated and free forms of the lysine. At this time point, the density for the Leu158 side chain has become noticeably weaker (Fig. 3B) compared to the apo enzyme (Fig. S5B) and *t* = 3 min structures (Fig. 3A), suggesting some increased motion of the side chain.

By *t* = 6 min, the acyl-enzyme intermediate is fully formed (Fig. 3C), with the polder map showing clear definition for the tail of NA-1-157 in a single conformation. The tail occupies a position against the side of the active site cleft, where it encroaches upon the Met213 side chain. In response, the side chain of Met213 becomes disordered and is observed (based on *F_o_–F_c_* density) in two conformations, both different from its position in apo-OXA-23 (Fig. 3D). Structural disorder in the Leu158 side chain is more evident at this time point, with two rotamers (closed and open) present in a 70%:30% ratio, respectively. This is in contrast to the OXA-23–meropenem complex where Val120 had moved to the open conformation.

We observed two water molecules near the inhibitor, one (W1) in the deacylating water pocket (DWP), hydrogen-bonded to Lys73^CO2^, and a second (W2) outside of the DWP, hydrogen bonded to the 6α-HE group and W1 (Fig. 3D). A third water (W3) was modeled deeper inside the CLP behind the Lys73^CO2^ side chain. The W1 is partially occupied (70%) and only present when Leu158 is in the open state. In its current location, it is ~6 Å from the scissile bond and is too far away to facilitate deacylation. In contrast to the current OXA-23–meropenem structure, the 6α-HE group is in an outward-facing orientation (17), where both the C61 and O62 atoms point away from Lys73^CO2^ (Fig. 3D), due primarily to steric clashes that would occur with the C5α-methyl if the 6α-HE were to face inward. Residual unmodeled *F_o_–F_c_* density peaks (red and green mesh; Fig. S7A) for the carboxylate group on Lys73 are more evident than at *t* = 4 min, reflecting a decrease in lysine carboxylation as time progresses. This residue was subsequently modeled and refined as a carboxylated lysine and a free lysine (with an associated water molecule, W4) with occupancies of 40% and 60%, respectively (Fig. S7B). No further increase in decarboxylation occurred after 10 min soaking, suggesting that the enzyme never becomes fully decarboxylated.

### Structural changes in OXA-23 due to carbapenem binding

**Fig 4:**
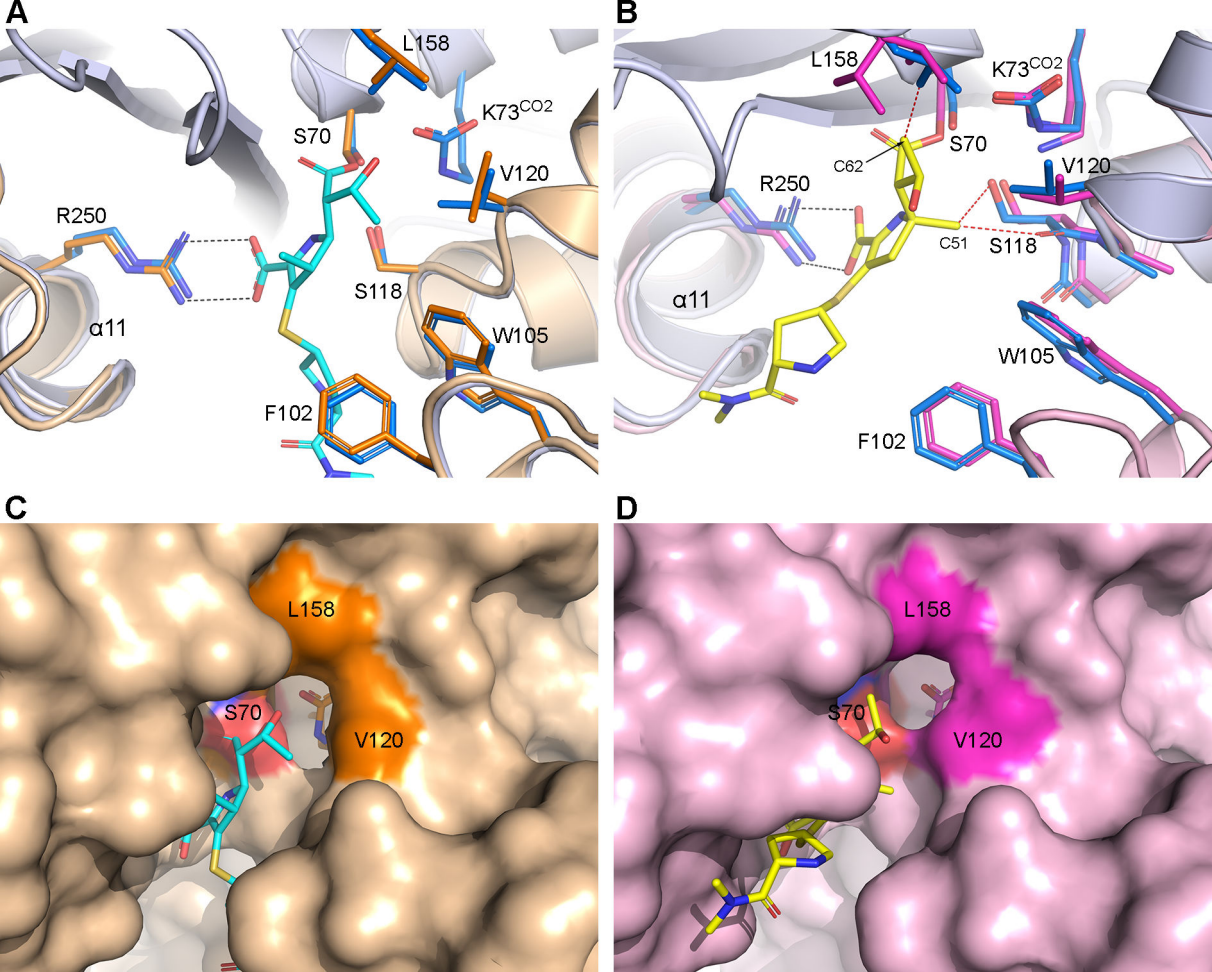
Comparison of the NA-1-157 and meropenem complexes of OXA-23. (**A**) Superposition of OXA-23–meropenem (orange ribbons and sticks) on apo-OXA-23 (light blue ribbons and blue sticks). Meropenem is shown in cyan. (**B**) Superposition of OXA-23–NA-1-157 (pink ribbons and magenta sticks) on apo-OXA-23. Steric clashes between NA-1-157 (yellow sticks), Leu158, and Ser118 in apo-OXA-23 are indicated by red dashed lines. Superpositions were based on residues 68–75 (encompassing the PASTFK motif, which includes the catalytic serine and lysine residues) and residues 207–211 (covering the KTG sequence motif on strand β5). (**C**) Molecular surface representation of the OXA-23–meropenem active site. Lys73^CO2^ is visible through the hole. (**D**) Molecular surface representation of the OXA-23–NA-1-157 active site.

The OXA-23–meropenem and OXA-23–NA-1-157 complexes were superimposed on the apo-OXA-23 structure (Fig. 4A and B). The presence of meropenem results in the outward movement of helix α11 by ~0.7 Å, primarily due to the interaction of the C3 carboxylate with the Arg250 side chain (Fig. 4A). On the opposite side of the active site cleft, residues Phe102 and Trp105 at the tip of the P-loop (12) move inward by about the same distance, driven by the formation of hydrophobic nonbonded contacts between the Phe102 side chain and the pyrrolidine ring. As noted above, the inward-facing 6α-HE rotamer results in the transition of the Val120 side chain to the open rotamer, which creates a hole in the hydrophobic cap (Fig. 4C), similar to what was observed in OXA-143 (27). This hole gives access to the internal CLP and is deemed essential for the ingress of a water molecule into the DWP (28, 29).

The formation of the OXA-23–NA-1-157 acyl-enzyme complex leads to different conformational changes in the active site compared to the meropenem complex. While the same outward movement of helix α11 (by ~1.0 Å) is observed for NA-1-157, the P-loop moves outward by ~0.8 Å (Fig. 4B), opposite to the movement observed in the meropenem complex. The presence of the C5α-methyl group in NA-1-157 (Fig. 1) generates steric pressure on Ser118, causing the α5–α6 loop (including Val120) to move outward by ~1 Å. However, the Val120 side chain remains in the closed conformation (Fig. 4B). The C62 atom exerts pressure on the Leu158 side chain (which is in the closed conformation), pushing it outward by ~0.7 Å relative to the equivalent position in apo-OXA-23. Together, these two movements of Val120 and Leu158 serve to widen the gap between them by ~1.2 Å. Since the hydrophobic interactions between these two residues are substantially reduced, this may explain why the Leu158 side chain is able to test the two rotameric conformations. A molecular surface calculation shows that the hydrophobic cap is open due to these concerted movements of the cap residues (Fig. 4D) (irrespective of whether Leu158 is open or closed), and water could enter the DWP. The presence of W1 in the crystal structure of the *t* = 6 min OXA-23–NA-1-157 complex (Fig. 3D) confirms this, although as noted earlier, this water molecule is too far from the scissile bond to trigger deacylation.

**Fig 5:**
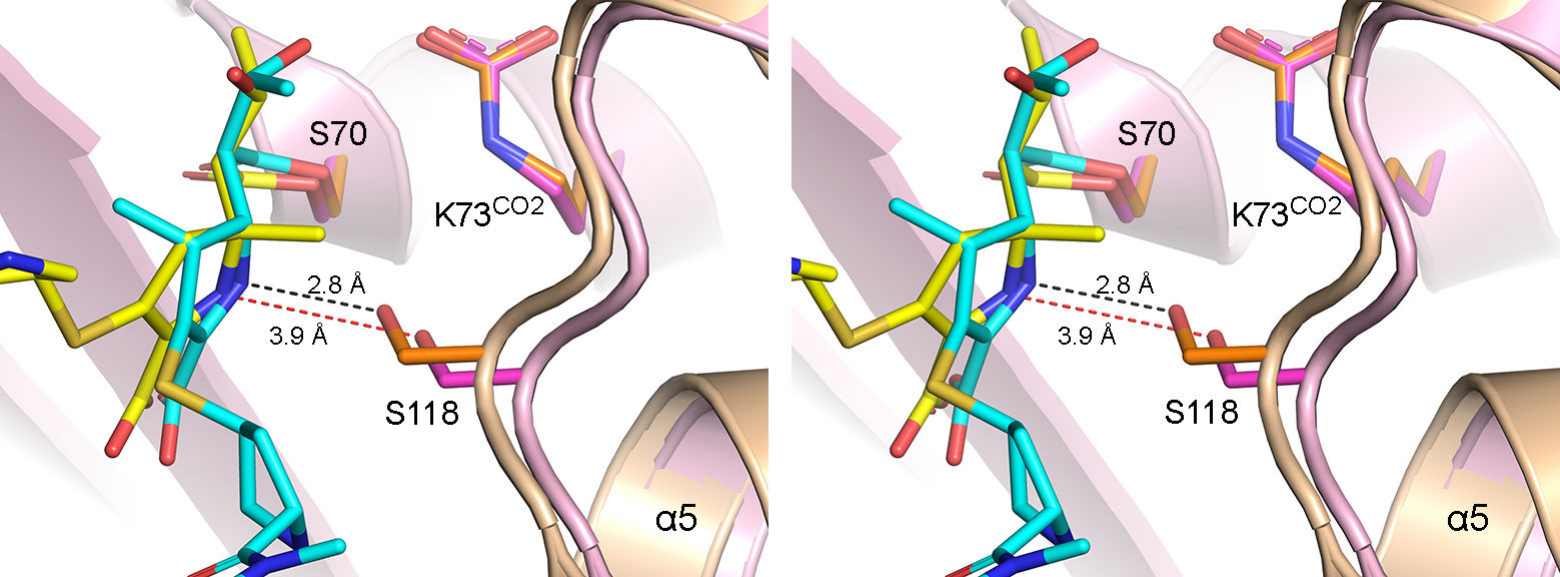
The OXA-23–meropenem and NA-1-157 complexes. Stereoview of the superposition of OXA-23–meropenem (orange ribbons and sticks) on OXA-23–NA-1-157 (pink ribbons and magenta sticks). Meropenem is shown in cyan and NA-1-157 in yellow. A hydrogen bond (black dashed line) is present between meropenem and Ser118, while the same interaction (red dashed line) is not observed in the inhibitor complex.

The outward movement of the α5–α6 loop not only opens a hole in the hydrophobic cap, but also positions the Ser118 side chain further from the pyrroline ring of NA-1-157 compared to the meropenem complex (Fig. 5). In the meropenem complex, there is a hydrogen bond (2.8 Å) between the Oγ atom of Ser118 and the N4 of the carbapenem; however, in the NA-1-157 complex, this hydrogen bond does not form (3.9 Å). The same observation was made recently in the NA-1-157 complex of OXA-58 at multiple time points (17), where loss of this bond was associated with perturbation of the proton shuttle necessary for acylation (Fig. S2). Since we also observed time-dependent decarboxylation of Lys73^CO2^ in OXA-23, the same mechanism is likely occurring, whereby the lack of connection between Ser118 and the N4 atom effectively disrupts the proton shuttle, ultimately leading to a buildup of positive charge on the carboxylated lysine which favors decarboxylation. The slower rate of NA-1-157 acylation (Table 2) could also be a consequence of this time-dependent decarboxylation of Lys73^CO2^.

### Molecular dynamics (MD) simulations

**Fig 6:**
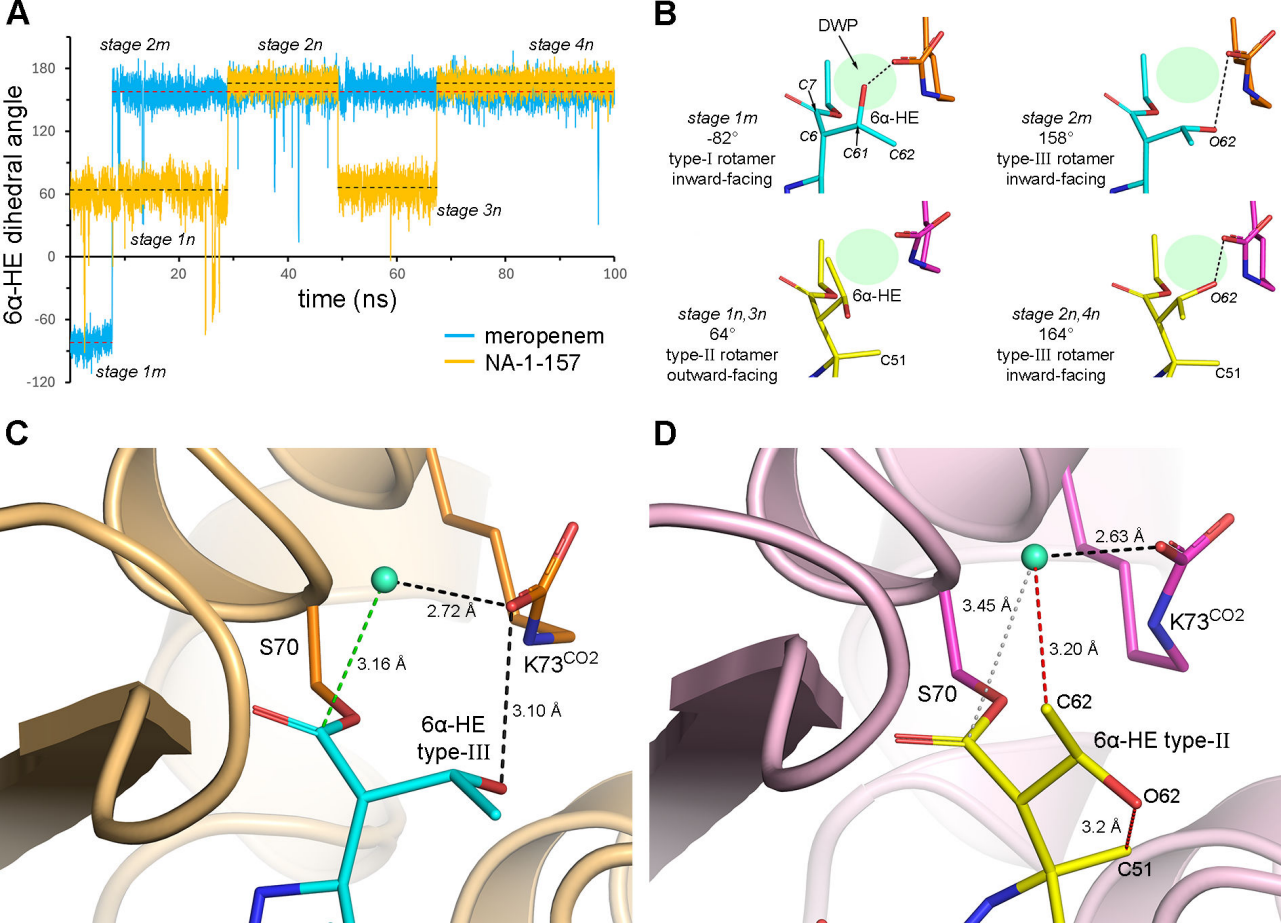
OXA-23–meropenem and OXA-23–NA-1-157 MD simulations. (**A**) Plot of the 6α-HE group dihedral angle for the OXA-23–meropenem (cyan) and NA-1-157 (yellow) complexes over the duration of the respective MD simulations. The different rotameric conformations of the 6α-HE group are indicated as a series of stages (1m and 2m for meropenem and 1n, 2n, 3n, and 4n for NA-1-157). (**B**) The 6α-HE group rotameric conformations for meropenem (blue sticks) and NA-1-157 (yellow sticks) from each stage of the plot in **A** are named according to the convention (31). The dihedral angle for each rotamer type is measured as the C7-C6-C61-C62 angle. The approximate location of the DWP is indicated in each structure. (**C**) A representative frame from the OXA-23–meropenem MD trajectory (at ~10 ns) showing a deacylating water molecule (light green sphere) coming to within 3.2 Å of the scissile bond (represented by the C7 atom of meropenem). The type-III rotamer of the 6α-HE group is indicated. The dotted green line shows the distance from the water molecule to the C7 atom. (**D**) A representative frame from the OXA-23–NA-1-157 MD trajectory (at ~3.9 ns) showing a deacylating water molecule (light green sphere) just inside the HPZD but unable to get closer to the scissile bond. The type-II rotamer of the 6α-HE group is indicated. The red dashed lines indicate steric clashes between the C62 and O62 atoms of the 6α-HE group, and the water molecule and C5α-methyl group, respectively. The dotted gray line shows the distance from the water molecule to the C7 atom.

To fully understand the dynamic nature of the OXA-23–meropenem and NA-1-157 complexes, we undertook 100 ns MD simulations of both. The complexes were stable for the duration of the simulations, as judged by the root mean square deviations (*rmsd*s) of the protein main chain relative to the initial model (1.8 and 2.0 Å for meropenem and NA-1-157, respectively). Analysis of the MD trajectory for the OXA-23–meropenem complex showed that the 6α-HE group, which has an initial C7-C6-C61-C62 dihedral angle of −82° (stage 1m, Fig. 6A), rotates to an alternate conformation (stage 2m) with a dihedral angle of 158° (on average) after ~7.7 ns. These two inward-facing rotameric forms for meropenem are designated here as type I and type III, respectively (Fig. 6B). In the type-I rotamer, the O62 atom is hydrogen bonded to Lys73^CO2^, a bond retained when the 6α-HE group transitions to the final type-III rotamer. In contrast, the 6α-HE group in the OXA-23–NA-1-157 complex has an initial dihedral angle of ~64° (stage 1n, Fig. 6A) in a type-II outward-facing rotamer (Fig. 6B). After ~29 ns, it moves inward (stage 2n, torsion angle 164° on average) into a type-III rotamer, similar to that observed in meropenem (Fig. 6B), and a hydrogen bond to Lys73^CO2^ forms. The 6α-HE group then oscillates between the type-II and type-III rotamers (stage 3n and stage 4n).

**Fig 7:**
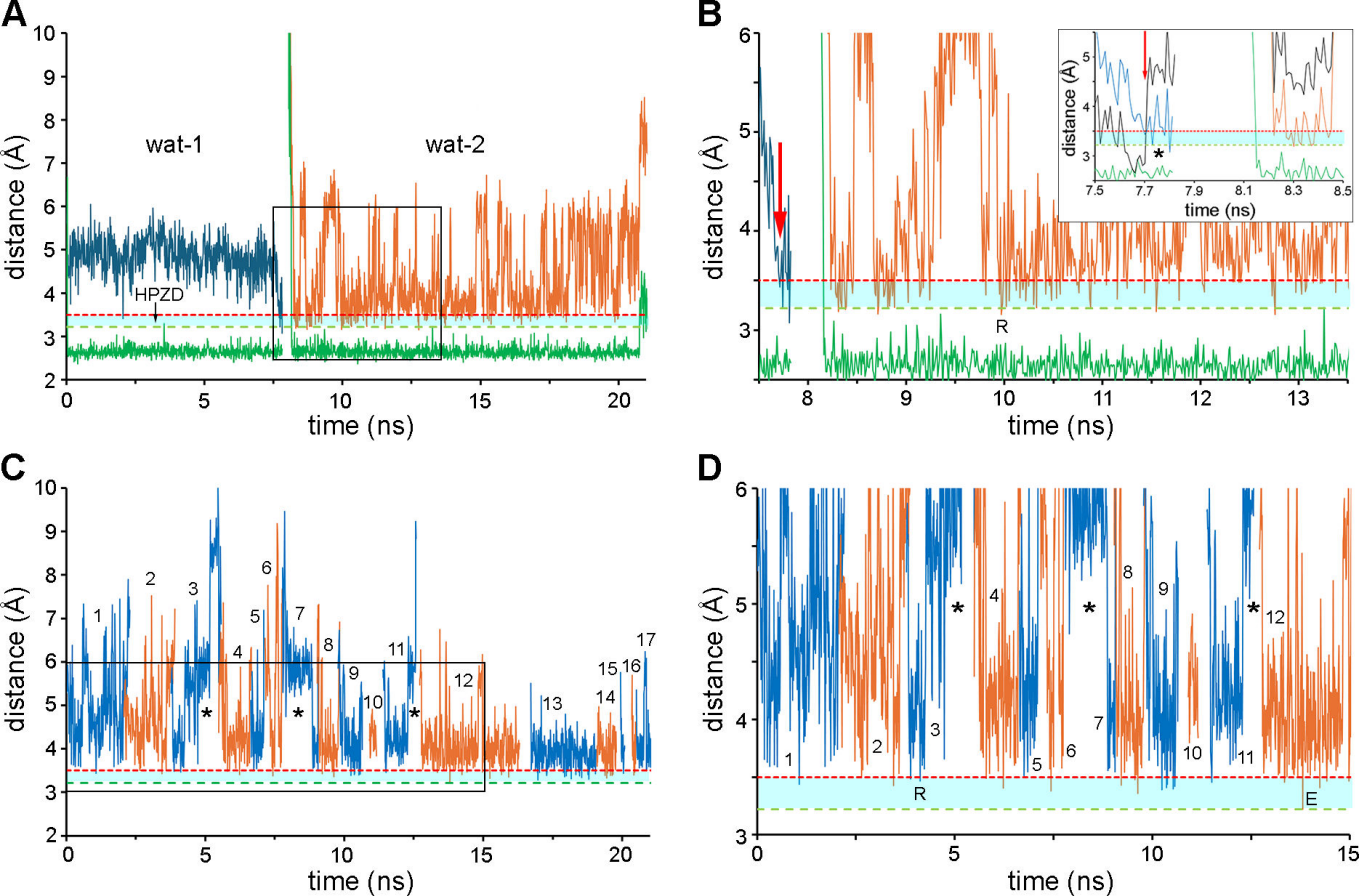
Analysis of MD simulations. (**A**) Composite plot of the distance to the C7 atom from two water molecules (wat-1 and wat-2, blue and orange traces, respectively) in the DWP of the OXA-23–meropenem MD trajectory. The distances of wat-1 and wat-2 from Lys73^CO2^ are also shown (green trace). The HPZD (pale blue shaded region) between 3.22 and 3.5 Å (green and red dashed lines, respectively) is shown. (**B**) Close-up view of the plot in **A** given by the region in the black box. The red arrow points to the time at which the 6α-HE group rotates from a type-I to a type-III rotamer. The point indicated by R is the frame taken as the representative for Fig. 6C. The inset shows a close-up between 7.5 and 8.5 ns. The black trace is the distance between the water and the O62 atom of the 6α-HE. The asterisk indicates two occasions where wat-1 comes into the HPZD to trigger deacylation. (**C**) Composite plot of the distance to the C7 atom from 17 consecutive water molecules in the DWP of the OXA-23–NA-1-157 MD trajectory. The traces for each water molecule are alternately colored blue and orange. The gaps indicated by the asterisks are frames when no water molecules entered the DWP. (**D**) Close-up view of the plot in **C**. Although some water molecules enter the zone, only one (indicated by E) comes to within 3.22 Å of the C7 atom and could trigger a deacylation event. The point indicated by R is the frame taken as the representative for Fig. 6D.

Since the major differences in the 6α-HE rotamer conformations between the two complexes occurred during the early stages of the simulations, we limited our detailed analysis of the MD trajectories to the first 21 ns. Inspection of the MD trajectory for the OXA-23–meropenem complex showed that two water molecules sequentially enter the DWP during this time and form a hydrogen bond with Lys73^CO2^ (green trace, Fig. 7A). The first water molecule (wat-1, blue trace, Fig. 7A) has an average distance of ~4.9 Å to the C7 atom (the target of nucleophilic attack by an activated water molecule), which is clearly too far away for effective deacylation. Only on one occasion (at ~2 ns) does it enter what we designate the high probability zone for deacylation (HPZD), where we consider the likelihood of effective nucleophilic attack to be higher. This area is between 3.22 Å (the C-O nonbonded contact distance) and 3.5 Å from the C7 atom. However, as this water molecule not only donates a hydrogen bond to Lys73^CO2^ but also accepts one from the O62 atom of the type-I 6α-HE rotamer (which decreases its nucleophilicity [31]), its activation for deacylation would be unlikely. When the 6α-HE group switches to the type-III rotamer (at ~7.7 ns), wat-1 immediately enters the HPZD to approach the C7 to within 3.2 Å (Fig. 7B). Crucially, at this moment, the hydrogen bond between wat-1 and the O62 atom breaks (black trace, Fig. 7B inset), but the bond to Lys73^CO2^ is retained (green trace, Fig. 7B inset), a condition ideal for water activation and deacylation of the OXA-23–meropenem complex. Similar conditions prevail for a second water molecule (wat-2, Fig. 6C), which enters the DWP at ~8.2 ns (orange trace, Fig. 7A and B). These observations highlight the dual role of the O62 atom in the type-I rotamer of the 6α-HE in preventing deacylation. Firstly, its proximity to the DWP sterically blocks water molecules from getting close enough to the scissile bond. Secondly, this atom can also donate a hydrogen bond to a water, thus nullifying activation by Lys73^CO2^. We conjecture that deacylation of meropenem may be retarded, while the 6α-HE group is in the type-I rotamer, and it is not until the group switches to the type-III rotamer (which is retained for the duration of the simulation) that an activated water can fully enter the HPZD for efficient nucleophilic attack.

Analysis of the first 21 ns of the OXA-23–NA-1-157 MD trajectory (Fig. 7C) showed that at least 17 water molecules enter and leave the DWP, but in contrast to meropenem, there are very few occasions where they enter the HPZD. Some come within 3.3–3.5 Å (Fig. 6D), and only one reaches within 3.22 Å of the C7 atom (Fig. 7D), but for most of the time, they are 4–5 Å away. During this stage of the simulation, the C62 atom of the type-II rotamer of the 6α-HE group is at the edge of the DWP and creates a steric barrier, limiting the approach of the water molecules, which would severely retard deacylation. This is consistent with our kinetic results, which show a very slow deacylation rate (Table 2).

### NA-1-157 complexes with β-lactamases

With this analysis of the OXA-23–NA-1-157 acyl-enzyme intermediate, we now have structural data on the inhibitor with three class D carbapenemases: OXA-48 (18), OXA-58 (17), and OXA-23. Since the OXA-48 complex was generated at acidic pH, Lys73 was decarboxylated, making deacylation impossible to investigate. In contrast, apo-OXA-23 and apo-OXA-58 were crystallized at pH 7.5 and 8.5, respectively, where the catalytic Lys73 is carboxylated and the enzymes are active, allowing direct structural comparison of their NA-1-157 complexes. Unlike OXA-23–NA-1-157, where there is only one monomer in the asymmetric unit, the OXA-58–NA-1-157 complex has four, with some monomers showing partial loss of CO_2_ and others full decarboxylation (17). Since the OXA-23 structure has both a carboxylated lysine and a free lysine, for comparison purposes, we chose OXA-58 monomer A, which also shows partial decarboxylation.

**Fig 8:**
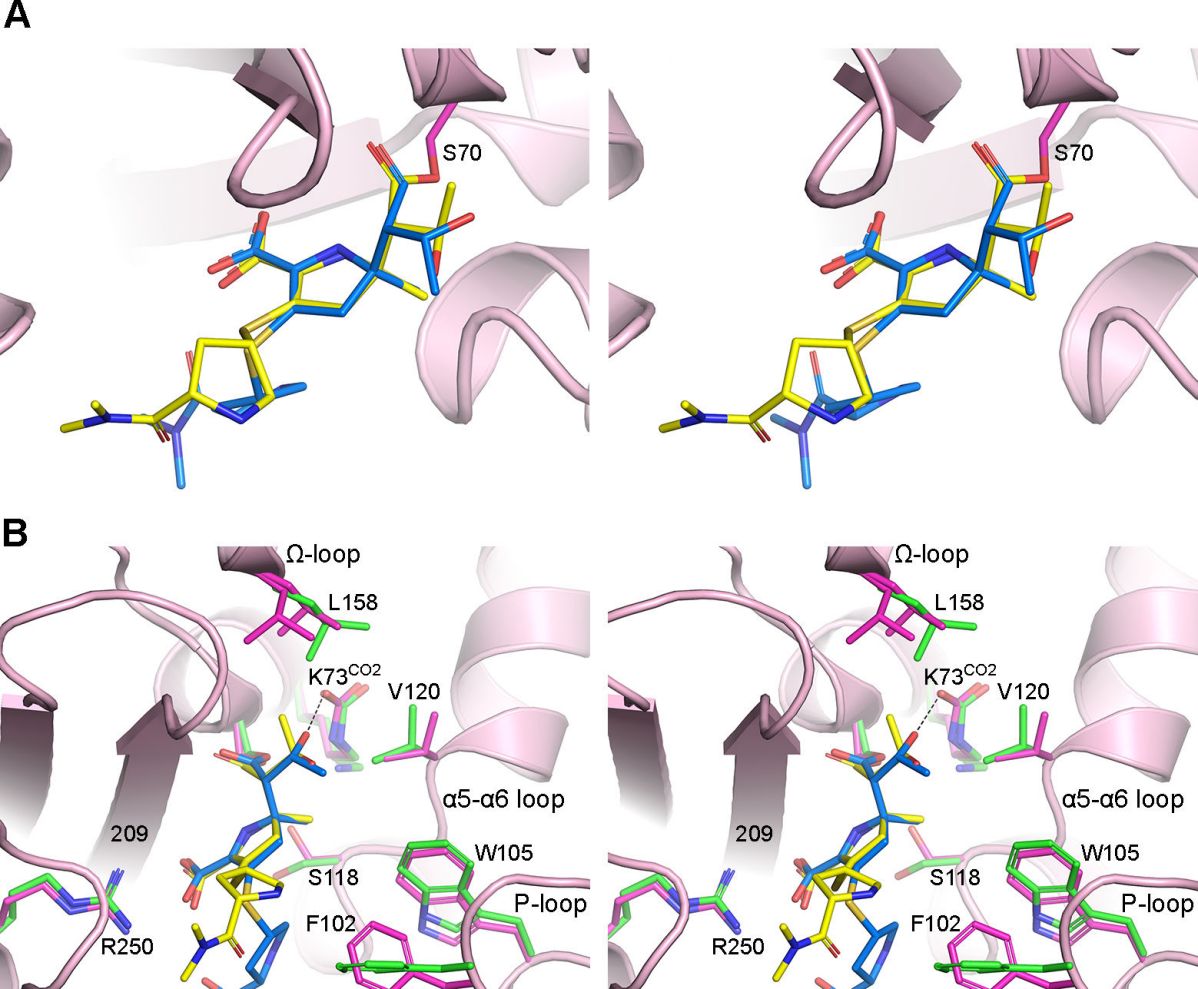
Superposition of the OXA-23 and OXA-58–NA-1-157 complexes. (**A**) Stereoview of the OXA-23 active site (pink ribbons) showing the bound NA-1-157 (yellow sticks) from *t* = 6 min. NA-1-157 from the *t* = 5 min OXA-58–NA-1-157 complexes is shown as blue sticks. The position of the pyrrolidine tail is variable. (**B**) Stereoview of the same superposition showing some of the residues lining the active site (magenta sticks for OXA-23 and green sticks for OXA-58). Residue 209 is a threonine in OXA-23 and a serine in OXA-58, so only the residue number is indicated.

In both complexes, NA-1-157 is covalently bound to the Ser70 side chain, and the pyrroline ring adopts the Δ^2^ tautomeric conformation. The core of the inhibitor is in roughly the same orientation in both complexes (Fig. 8A), and the C3 carboxylate groups make identical interactions with residue 209 (Thr209 in OXA-23 and Ser209 in OXA-58) and Arg250. There is, however, a major difference in the rotameric conformation of the 6α-HE groups: type-II in OXA-23 and type-III in OXA-58 (Fig. 8A). In the latter case, the O62 atom donates a long hydrogen bond (~3.4 Å) to the partially occupied Lys73^CO2^ (Fig. 8B). The available structural data do not allow us to say with any certainty why different conformational states are preferred in the two enzymes.

There are also noticeable differences in the P-loop, α5-α6 loop, and Ω-loop residues between the two enzymes (Fig. 8B). The major point of deviation is the relative positions of the hydrophobic cap residues (Val120 and Leu158) in response to inhibitor binding. While in both structures we observed an increase in the distance between these residues, the resulting gap is significantly wider in OXA-23, and our MD results showed that this allows water molecules to enter the DWP of the enzyme. To investigate the dynamics of the 6α-HE group of NA-1-157 in OXA-58 and whether water can also enter the DWP as a result of the observed widening (17), we ran a 100 ns MD simulation on the OXA-58–NA-1-157 complex containing Lys73^CO2^. Analysis of the MD trajectory showed that in contrast to the OXA-23 simulation, the 6α-HE group remains in the type-III rotamer for the duration of the simulation, except for several short-lived transitions to type-I and type-II states (Fig. S8A). A strong hydrogen bonding contact (~2.7 Å on average) between O62 and the Lys73^CO2^ is present for the first 25 ns, but this interaction lengthens to ~4 Å (on average) until the end of the simulation (Fig. S8B), due to a small swing of the Lys73^CO2^ side chain away from the scissile bond by ~0.8 Å. Furthermore, the OXA-58 MD simulation does indeed show that several water molecules come into the putative DWP between the scissile bond and Lys73^CO2^. However, the constant presence of the O62 atom of the 6α-HE group within the DWP prevents them from approaching closer than ~4 Å of the C7 atom (Fig. S9A and B).

### Conclusions

In this manuscript, we describe the interaction of the novel carbapenem NA-1-157 with the major *A. baumannii* CHDL OXA-23. The compound had significantly lower MICs against clinical isolates of MDR *A. baumannii* producing OXA-23 and other class D carbapenemases, compared to the commercial carbapenems imipenem and meropenem. Kinetic experiments demonstrated that NA-1-157 is an inhibitor of OXA-23, where the majority of acylation proceeds inefficiently, while deacylation is severely impaired. These experiments, in conjunction with MS results, also allow us to propose that the observed slow phase of acylation for OXA-23 and other enzymes is caused by the formation of a reversible covalent pre-acylation intermediate(s).

Structural studies and MD simulations showed that inhibition of the OXA-23 by NA-1-157 is due to a combination of two molecular mechanisms resulting from the presence of the C5α-methyl group in the compound. One involves partial decarboxylation of the catalytic lysine residue due to perturbation of the proton shuttle. The second mechanism is restricted access of the deacylating water to the scissile bond. Comparison of the NA-1-157 interaction with other carbapenemases demonstrated that there are differences, even between closely related enzymes, such as OXA-23 and OXA-58. While both enzymes have restricted access of water to the scissile bond due to the encroachment of the C62 (in OXA-23) or O62 (in OXA-58) atoms into the DWP, the extent to which lysine decarboxylation occurs is dissimilar. In contrast to complete decarboxylation of the catalytic lysine in OXA-58, only partial lysine decarboxylation occurs in OXA-23. Thus, the varying contributions of these processes (lysine decarboxylation and water access) in these two enzymes fine-tune the molecular mechanisms of their inhibition by NA-1-157. The observed variations caution against generalizations regarding the potential mechanisms of interaction of novel antibiotics/inhibitors, even with closely related enzymes, and highlight the need to study each system independently.

## MATERIALS AND METHODS

### Antibiotic susceptibility testing

MDR *A. baumannii* clinical isolates were from the *Acinetobacter baumannii* and Gram-negative carbapenemase detection panels from the CDC & FDA AR Isolate Bank (20). MICs were determined by the broth microdilution method according to the CLSI guidelines (32).

### Cloning and protein purification

The 5’-truncated (54 bp) *E. coli*-optimized *bla*_OXA-23_ gene (28) was cloned into the *Nde*I and *Hind*III sites of pET24a(+) (Invitrogen) and transformed into *E. coli* BL21 (DE3). OXA-23 was expressed as previously described (28) and purified from cell lysates in 25 mM HEPES pH 7.0 using two ion exchange columns (Bio-Rad) equilibrated with the same buffer. First, OXA-23 was isolated from the flow through of a DEAE column and subsequently eluted from a High S column with a linear gradient of 0–1 M NaCl. Fractions were collected and analyzed for purity by SDS-PAGE. For kinetic and X-ray crystallography studies, the enzyme was concentrated and dialyzed against either 100 mM sodium phosphate pH 7.0 or 25 mM HEPES pH 7.5, respectively. For longer storage at −80°C, purified OXA-23 was aliquoted in 10% glycerol containing buffer.

### Kinetic studies

Data were collected using either a Cary 60 spectrophotometer (Agilent) or an SFM-300 stopped-flow mixing system (Bio-Logic). Reactions were carried out at 22°C in 100 mM sodium phosphate pH 7.0 containing 50 mM NaHCO_3_, unless otherwise stated. All measurements were completed in at least triplicate. Data analysis was performed nonlinearly using Prism 10 (GraphPad Software, Inc.).

#### Steady-state reactions

Reactions containing various concentrations (25–100 µM) of NA-1-157 and 0.2 mg/mL BSA were initiated by the addition of OXA-23 (1–10 µM). The change in absorbance was monitored at 308 nm, and the progress curves were corrected for nonspecific hydrolysis of NA-1-157 (19).

#### Determination of the dissociation constant *K*_i_ and inactivation parameters *k*_NA-1-157_ and *K*_I_

These parameters were evaluated using a competition experiment with nitrocefin (Δε = 500 nm; = 15,900 M^−1^cm^−1^). Reactions consisting of NA-1-157 at various concentrations (0–8 µM), 400 µM nitrocefin, and 0.2 mg/mL BSA were initiated by the addition of OXA-23 (0.21–0.84 nM). The progress curves were fitted to the time-dependent equation for slow-binding inhibitors (21) to determine the initial velocities and *k*_inter_ values. To calculate *K*_*i*_, the initial velocities were plotted versus the concentration of NA-1-157, and the data were fitted to the Morrison equation (21). To determine *k*_NA-1-157_ and *K*_I_, the *k*_inter_ values were plotted versus the concentration of NA-1-157, and the data were fitted to a two-step irreversible inhibition model, as previously described (19).

#### Evaluation of the acylation rate constants *k*_2_

Reactions under single turnover conditions containing NA-1-157 (5–25 µM) were initiated by the addition of OXA-23 (25–250 µM), and the change in absorbance was monitored at 308 nm. Progress curves of the biphasic reactions were fitted as previously described to determine *k*_2 fast_ and *k*_2 slow_ (19).

#### Evaluation of the deacylation rate constant *k*_3_

The jump dilution method (21) with discontinuous sampling was used for deacylation experiments. A reaction containing 10 µM NA-1-157, 3 µM OXA-23, and 0.2 mg/mL BSA was incubated for 20 min at 22°C and passed through two successive Zeba 0.5 mL columns (7 kDa molecular weight cutoff, Thermo Scientific) to remove excess compound. Next, the reaction was diluted 4,000-fold into buffer containing 0.2 mg/mL BSA. Aliquots were removed at various time points, and the enzyme activity with 500 µM nitrocefin was measured. Control reactions in the absence of NA-1-157 were also performed under the same conditions. The percentage of activity was plotted versus time, and the data were analyzed as previously described (19).

### ESI-LC/MS

Reactions containing NA-1-157 (20 µM) and OXA-23 (2 µM) in 100 mM sodium phosphate pH 7.0, 50 mM NaHCO_3_ were quenched after 10 s with 10% acetonitrile/1% formic acid and stored at −80°C until analysis. The control reaction contained no NA-1-157. For competition experiments, aliquots were removed after 10, 30, 90, and 300 s and mixed with 2.5 mM PQ-1-219 (MW = 298 g/mol). They were immediately quenched and stored as described above. The control reaction contained no NA-1-157. ESI-LC/MS analysis was carried out using a DIONEX Ultimate 3000 system connected to a Bruker micrOTOF-QII mass spectrometer in positive-ion mode, utilizing Hystar 4.2 SR2 software, and data were evaluated as previously described (19).

### Crystallography

Crystals of OXA-23 were grown by sitting drop in Intelliplates from 0.2 M Li_2_SO_4_, 0.1 M HEPES pH 7.5, 25% PEG3350 (SG1 screen, #1-42, Molecular Dimensions). The crystals belonged to space group P4_2_2_1_2 with cell dimensions 83.3 × 83.3 × 85.9 Å, diffracting to ~1.3 Å resolution. Since the presence of tightly bound sulfate or phosphate anions in the active site of enzymes can be detrimental to the binding of substrates (33–38), the crystallization buffer was exchanged for 0.1 M imidazole (pH 7.6), 34% MPD, and 25% PEG3350 using established protocols (33, 37) to allow meropenem and NA-1-157 to compete effectively with the sulfate in subsequent soaking experiments. Apo-OXA-23 crystals soaked in this stabilizing buffer showed no deterioration in diffraction quality. The 34% MPD provided adequate cryoprotection with no additional additives required. Data were collected from a single apo-OXA-23 crystal flash-cooled in liquid nitrogen. The images were processed and scaled with XDS (39) and AIMLESS (40) to 1.4 Å resolution. The final data collection statistics are given in Table S2. The structure was solved by molecular replacement using the apo-OXA-23 structure at pH 7.0 (PDB code 4JF6) as the starting model. The structure was refined with *phenix.refine* (41), and the final model comprises a single monomer in the asymmetric unit. Refinement statistics are given in Table S2.

An OXA-23–meropenem complex was prepared by soaking apo-OXA-23 crystals in a 20 mM meropenem solution in stabilizing buffer for 6 min before flash-cooling in liquid nitrogen. Diffraction data were collected and processed as described above for apo-OXA-23. Time-resolved NA-1-157 binding experiments were conducted by soaking apo-OXA-23 crystals for 3, 4, 6, and 10 min in a 10 mM NA-1-157 solution in stabilizing buffer. Diffraction data were collected and processed as described above. All complex structures were solved by molecular substitution (Fourier synthesis) using the apo-OXA-23 structure as a starting model for refinement, since the complexes have the same unit cell and space group as the apo enzyme. Data collection and refinement statistics for the meropenem complex are given in Table S2 and for the NA-1-157 complexes in Table S3.

MD simulations were performed in triplicate for 100 ns on cyrstal structures of the OXA-23–meropenem, the 6-min OXA-23–NA-1-157, and monomer A of the 5-min OXA-58–NA-1-157 complexes (9D7A), all with fully occupied Lys73^CO2^ . The simulations were run with Desmond (42) from the Schrodinger 2019-2 release, as described previously (16, 19). Analyses of the resultant trajectories were carried out with Maestro (Schrodinger). Maestro and Desmond were run on the SHERLOCK 3.0 HPC cluster at Stanford University.


$$E+I\underset{k_{-1}}{\overset{k_{1}}{⇌}}EI\overset{k_{2}}{⟶}E-I\overset{k_{3}}{⇢}E+P$$

Scheme 1. Minimal reaction mechanism for inhibition of OXA-23 by NA-1-157. *E* represents the enzyme, *I* is the inhibitor, *EI* is the noncovalent Michaelis complex and any other species formed prior to acylation, *E-I* is the covalent acyl-enzyme species, and *P* is the hydrolyzed product. The dashed arrow indicates that deacylation takes place only very slowly. The rate constant *k*_2_ includes both *k*_2 fast_ and *k*_2 slow_. The pre-acylation covalent reversible species described in the text is not shown.

## Data Availability

The apo-OXA-23 and its complexes with NA-1-157 and meropenem structure factors and atomic coordinates have been deposited in the PDB with PDB codes 9NSW, 9NSX, 9NSY, 9NSZ, and 9NT0. The authors will release the atomic coordinates and experimental data upon article publication.

## SUPPLEMENTAL MATERIAL

Supplemental material aac.00918-25-s0001.docx Fig. S1 to S9; Tables S1 to S3.

ASM does not own the copyrights to Supplemental Material that may be linked to, or accessed through, an article. The authors have granted ASM a non-exclusive, world-wide license to publish the Supplemental Material files. Please contact the corresponding author directly for reuse.
