# Supplementary material for: Dual mechanism of the OXA-23 carbapenemase inhibition by the carbapenem NA-1-157
Source: Antimicrob Agents Chemother. 2025 Aug 20;69(10):e00918-25. doi: 10.1128/aac.00918-25 (PMC12486850; doi:10.1128/aac.00918-25)
Supplement: Supplemental material — Fig. S1 to S9; Tables S1 to S3. [file aac.00918-25-s0001.docx]

**Dual Mechanism of the OXA-23 Carbapenemase Inhibition by the Carbapenem NA-1-157**

Marta Toth^a^, Nichole K. Stewart^a^, Pojun Quan^b^, Md Mahbub Kabir Khan^b^, Jonathan Cox^b^, John D. Buynak^b,#^, Clyde A. Smith^c,d,#^, and Sergei B. Vakulenko^a,#^

^a^ Department of Chemistry and Biochemistry, University of Notre Dame, Notre Dame, IN 46556, USA

^b^ Department of Chemistry, Southern Methodist University, Dallas, TX 75275, USA

^c^ Stanford Synchrotron Radiation Lightsource, Stanford University, Menlo Park, CA 94025, USA

^d^ Department of Chemistry, Stanford University, Stanford, CA 94305, USA

^#^ To whom correspondence should be addressed:

Prof. John D. Buynak, ph: 214-768-2484, Fax: 214-768-4089, E-mail: jbuynak@smu.edu

Dr. Clyde A. Smith, ph: 650-926-8544, Fax: 650-926-3292, E-mail: csmith@slac.stanford.edu

Prof. Sergei B. Vakulenko, ph: 574-631-2935, Fax: 574-631-6652, E-mail: svakulen@nd.edu

**
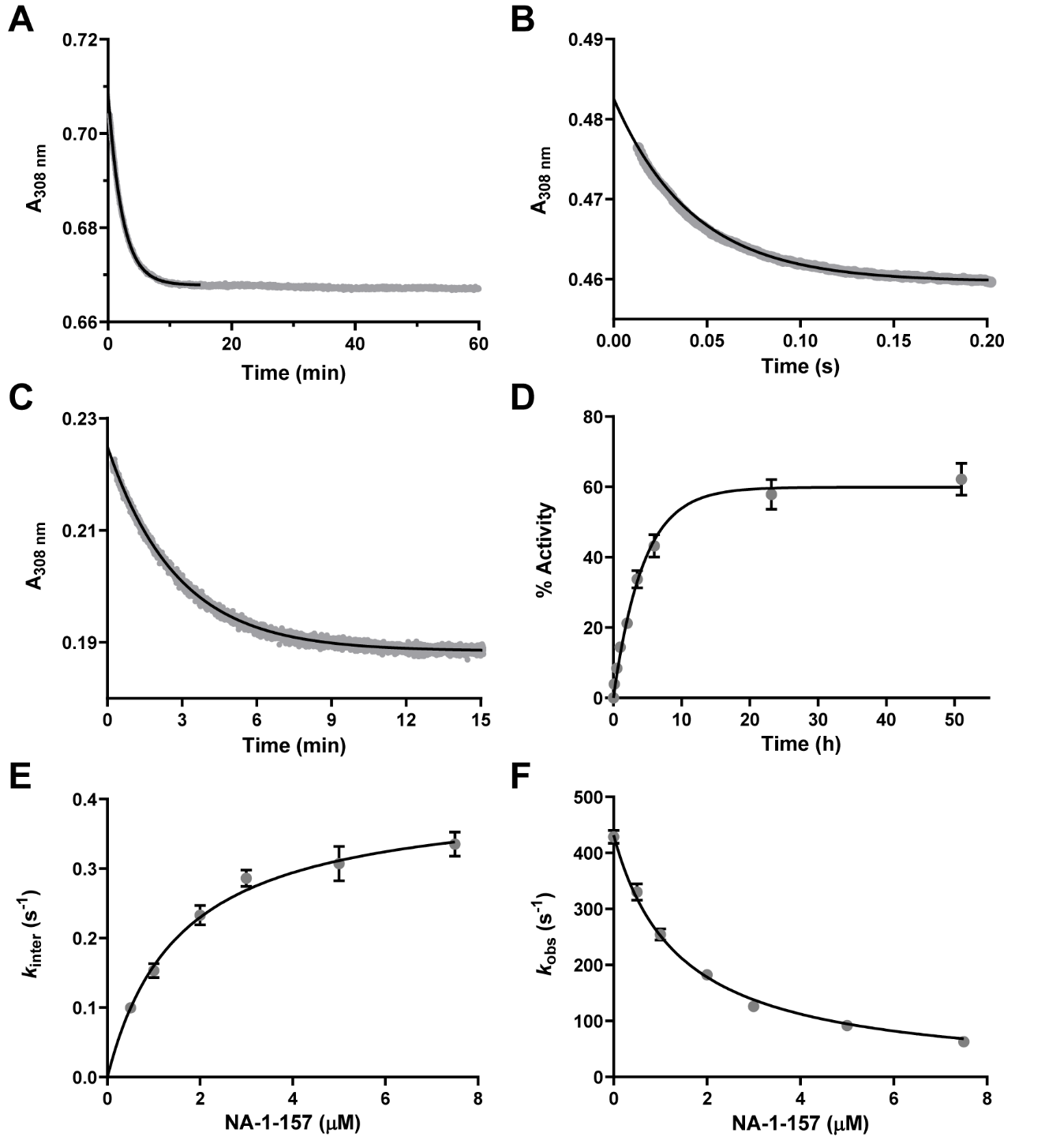
**

**Figure S1. Kinetics of the interaction between OXA-23 and NA-1-157.** **(A)** Representative progress curve under steady-state conditions showing inhibition of OXA-23 by NA-1-157. **(B)** Single turnover time course with 10-fold molar excess OXA-23 showing the fast phase of acylation. **(C)** Single turnover time course with 10-fold molar excess OXA-23 showing the slow phase of acylation. **(D)** Time course for recovery of OXA-23 activity after incubation with NA-1-157 and subsequent jump dilution. **(E)** Plot of *k*_inter_ values versus concentration of NA-1-157. **(F)** Plot of hydrolysis rate of nitrocefin versus concentration of NA-1-157. In all panels, the lines of best fit are shown in black. The error bars reflect the standard deviation of the measurements.

**
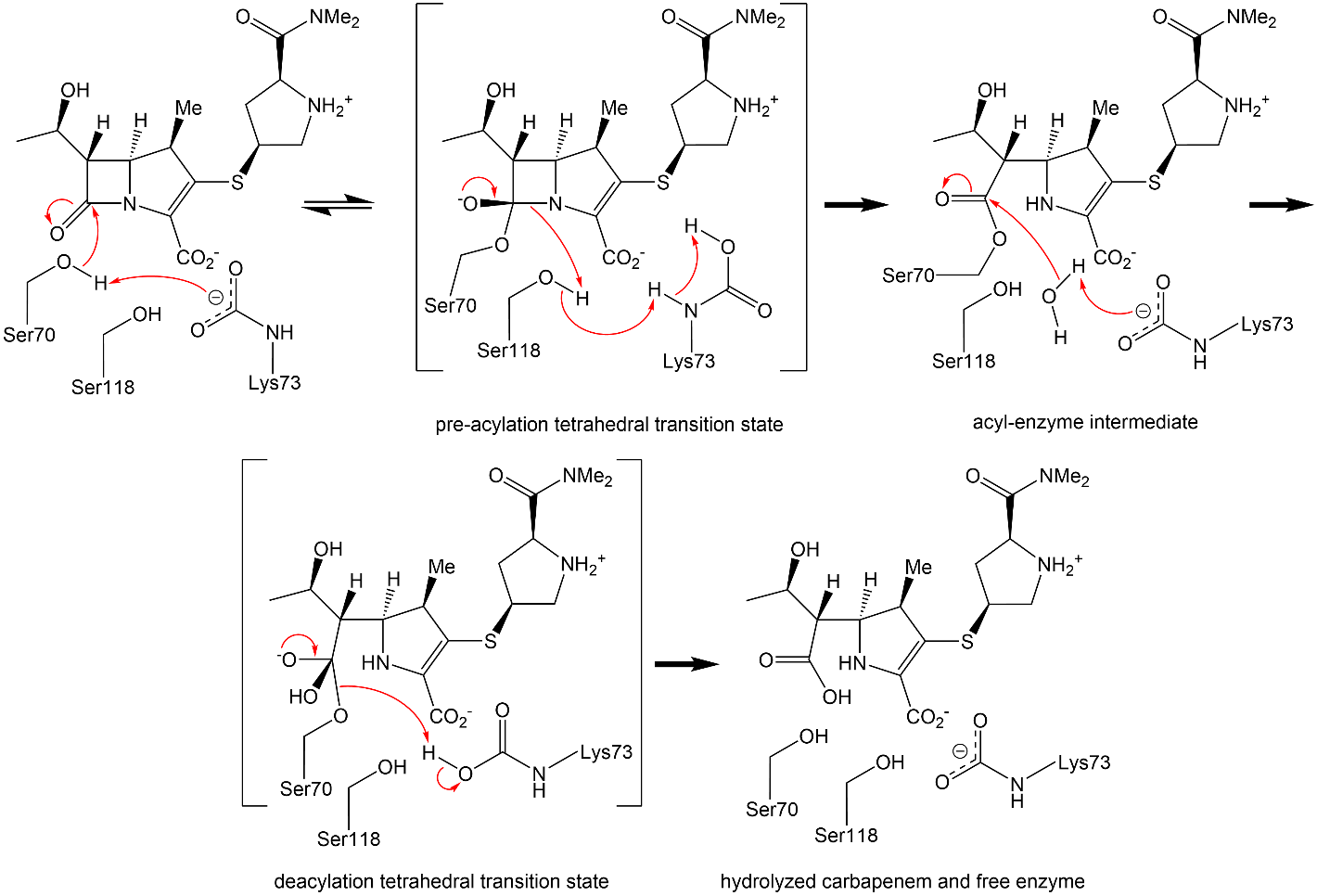
**

**Figure S2. General mechanism for hydrolysis of carbapenems by CHDLs.** The reaction for meropenem is shown. In the case of NA-1-157, the reaction beyond the acyl-enzyme intermediate is severely impaired.

**Figure S3. Evaluation of** **OXA-23-NA-1-157 complexes by mass spectrometry.** **(A)** Mass spectra of apo-OXA-23 (brown, 28,950 Da) alone and its complexes formed after incubation with either an excess of NA-1-157 (black, 29,334 Da and 29,290 Da; the latter resulting from loss of either CO_2_ or the 6α-HE) or PQ-1-219 (blue, 29,249 Da, 29,205 Da, and 29,163 Da; the last two resulting from loss of either CO_2_ or the 6α-HE or both, respectively). The major peak of OXA-23 and its complexes are labelled on the spectra with their masses in Da. **(B)** Mass spectra of the OXA-23 complexes formed after 10 s (blue), 90 s (orange), and 300 s (black) of incubation with a 10-fold excess of NA-1-157, followed by chasing with an excess of PQ-1-219.


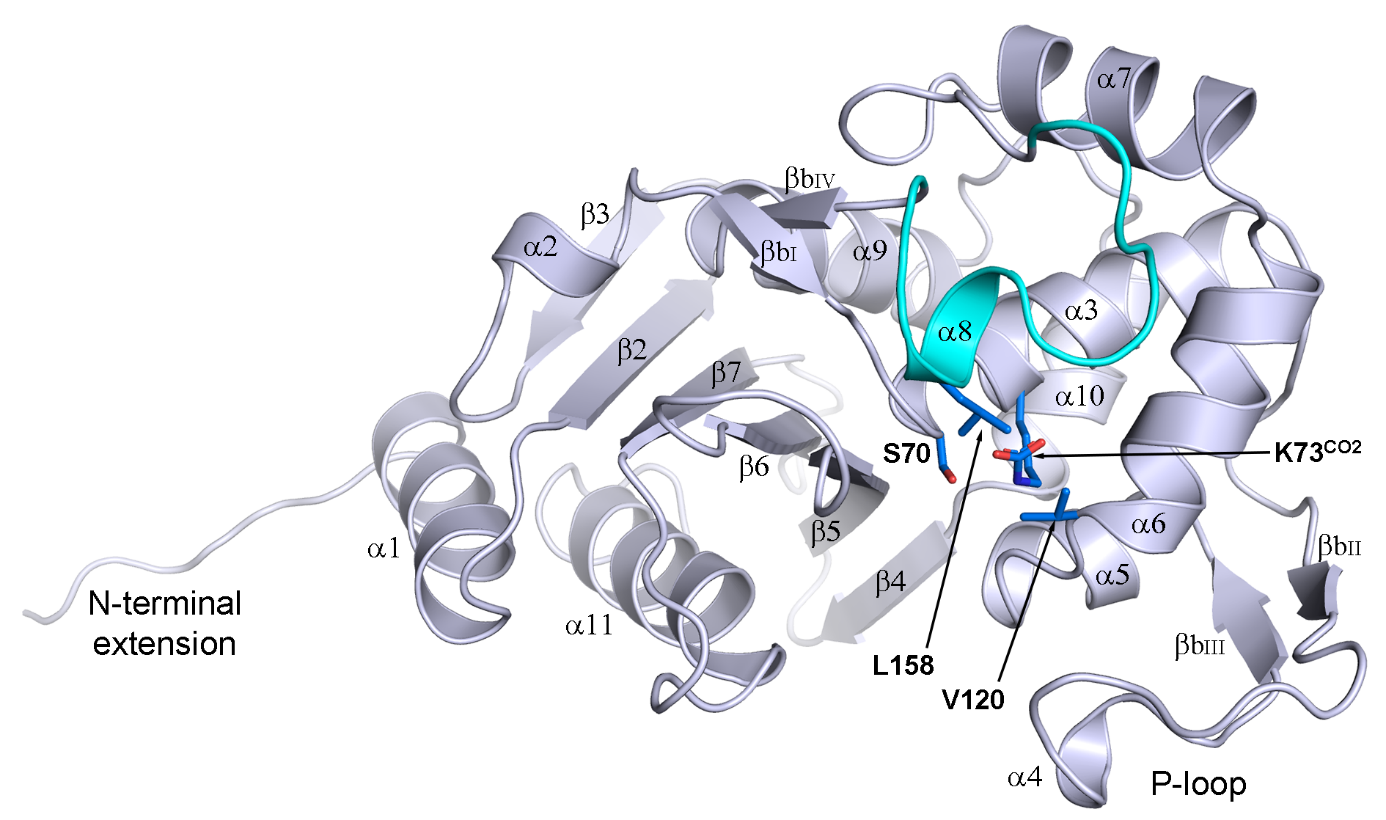


**Figure S4. Apo-OXA-23 structure.** Secondary structural elements are labeled. The Ω-loop is colored cyan, and side chains of four residues (Ser70, Lys73^CO2^, Val120, and Leu158) are shown as blue sticks. The long P-loop between helices α3 and α5 is indicated. The N-terminal extension of eight residues extends from the structure as shown on the left.


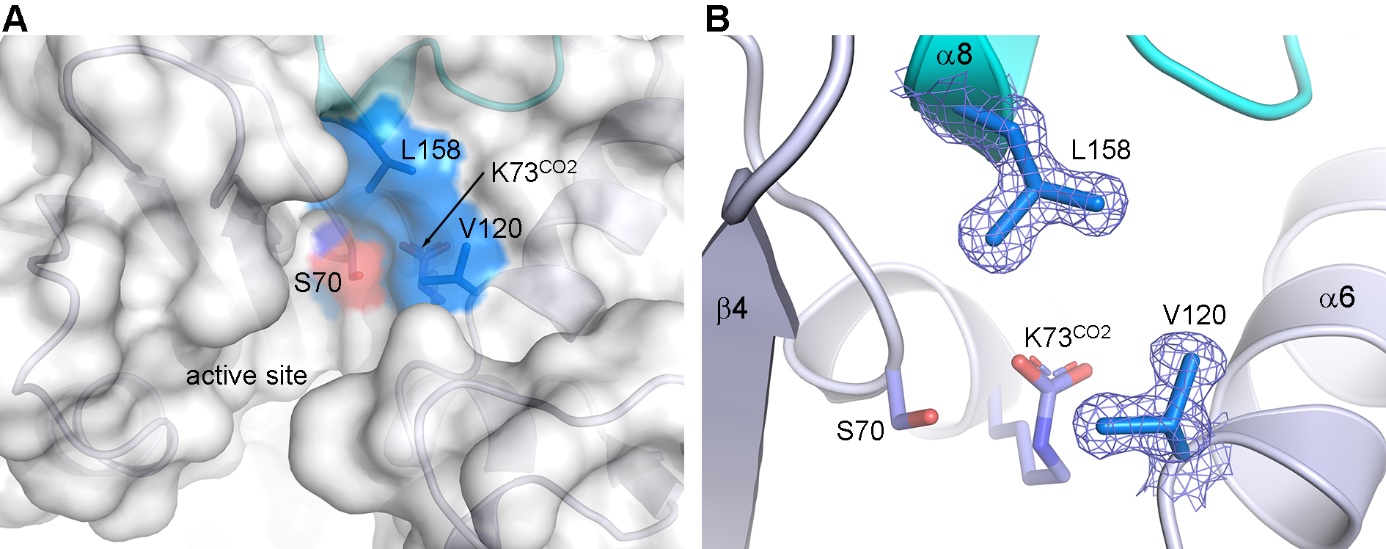


**Figure S5. The hydrophobic cap in apo-OXA-23. (A)** Semi-transparent molecular surface representation in the vicinity of the active site showing the closed surface formed by the side chains of Val120 and Leu158. The locations of the catalytic Ser70 and Lys73^CO2^ residues are indicated. **(B)** Final 2*F_o_-F_c_* electron density (blue mesh, 1.2 σ) for the Val120 and Leu158 side chains.


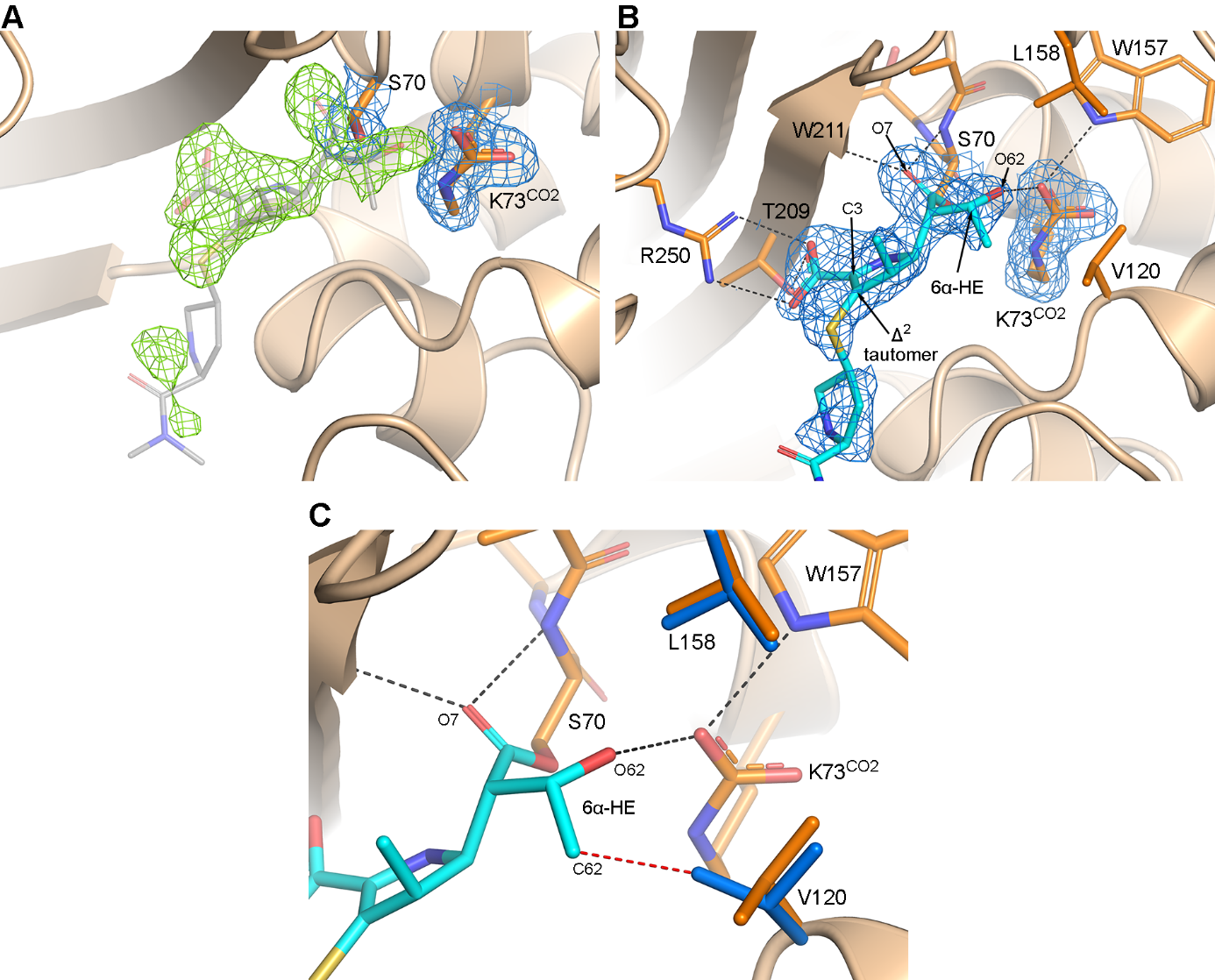


**Figure S6. The OXA-23-meropenem complex. (A)** The active site of the complex (orange ribbons and sticks) showing residual *F_o_-F_c_* density (green mesh, 3.5 σ) attached to the catalytic Ser70. The Ser70 and K73^CO2^ side chains are shown in 2*F_o_-F_c_* density (blue mesh, 1.2 σ). The location of meropenem from the final refined structure is shown as semi-transparent gray sticks. **(B)** Final 2*F_o_-F_c_* density (blue mesh, 1.2 σ) for meropenem (cyan sticks). Hydrogen bonds are shown as black dashed lines. **(C)** The hydrophobic cap residues (Val120 and Leu158) from apo-OXA-23 (blue sticks) superimposed on the OXA-23-meropenem complex. The potential close contact between the C62 atom and the Val120 side chain in the apo structure is indicated by the red dashed line.


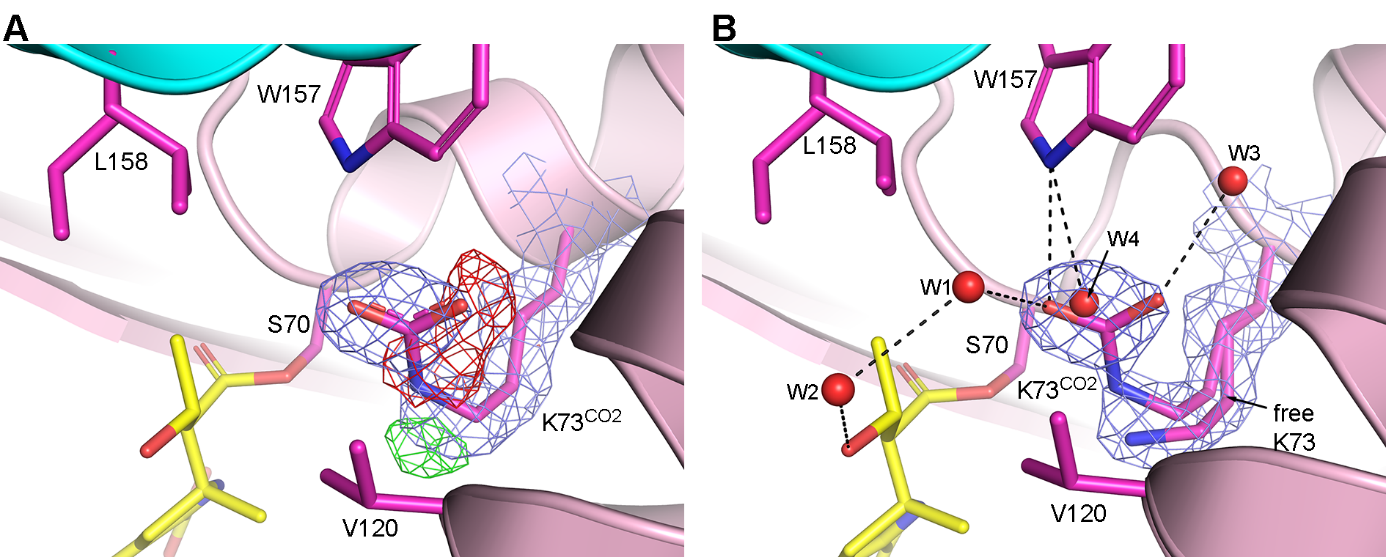


**Figure S7. The OXA-23-NA-1-157 acyl-enzyme structure at the 6 min time point. (A)** Refined 2*F_o_-F_c_* electron density for the Lys73^CO2^ side chain (blue mesh, 1.2 σ) is shown. A residual *F_o_-F_c_* map (green mesh, +3 σ; red mesh, -3 σ) is superimposed. The negative red peak suggests that there is a lack of electron density at the Nζ atom and one of the oxygen atoms of the carbamate moiety, and the positive green peak denotes the presence of a partially-occupied free lysine. The lack of negative density for the whole of the carbamate suggests the presence of a water in conjunction with the free lysine. **(B)** Final active site structure, showing the 2*F_o_-F_c_* density (blue mesh, 1.2 σ) for a partially-occupied Lys73^CO2^ and a free lysine and associated water (W4). The locations of three other water molecules (W1, W2, and W3) are also indicated. NA-1-157 is shown as yellow sticks in both panels.


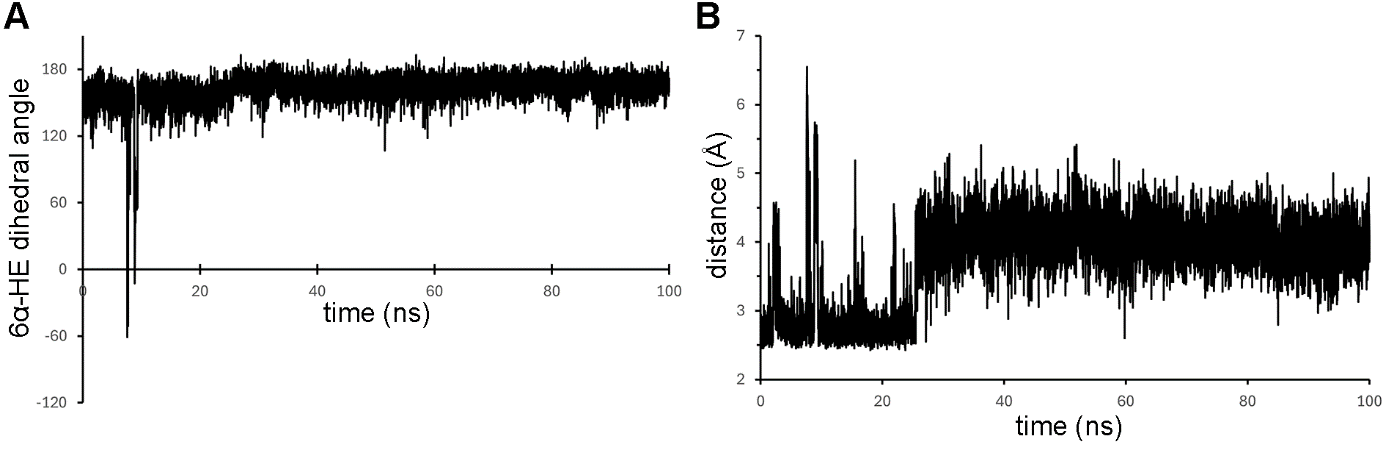


**Figure S8. MD simulation of the OXA-58-NA-1-157 acyl-enzyme complex. (A)** Plot of the 6α-HE group dihedral angle for the OXA-58-NA-1-157 complex over the duration of the MD simulation. A type-III rotamer of the 6α-HE group is maintained throughout the trajectory. **(B)** Plot of the distance between the O62 atom of the 6α-HE group and one of the carboxylate oxygen atoms on Lys73^CO2^.

**
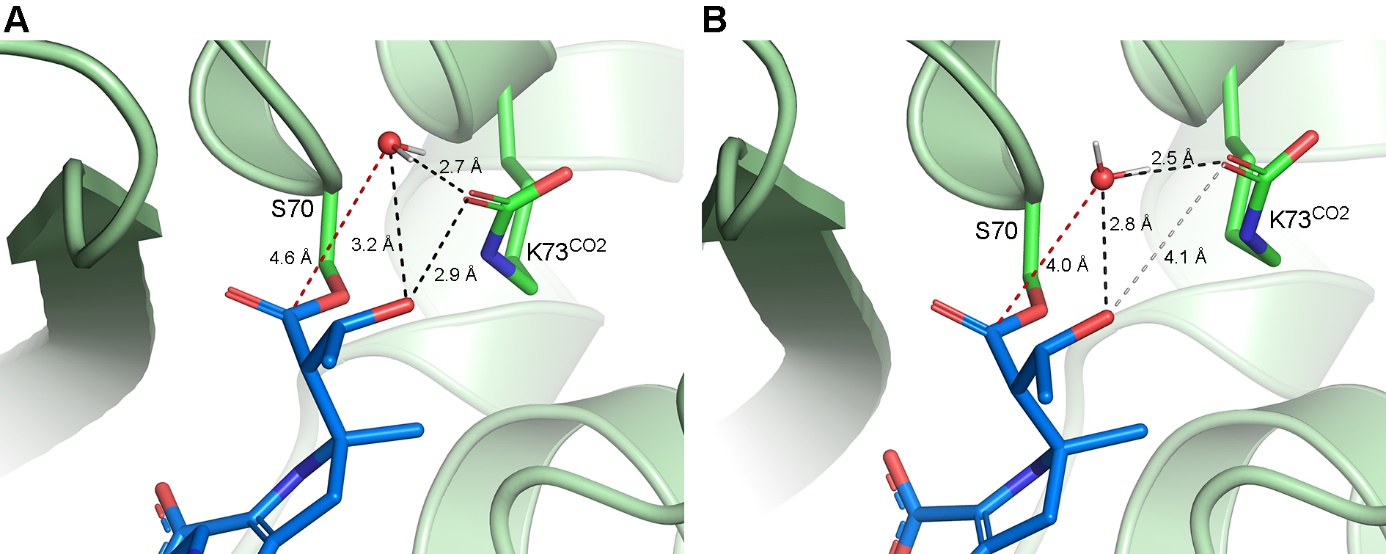
**

**Figure S9. Frames from the MD simulation of the OXA-58-NA-1-157 complex. (A)** Representative frame from t < 25 ns of the trajectory, when the hydrogen bond between the O62 atom and the Lys73^CO2^ side chain is intact. **(B)** Representative frame from t > 25 ns, after the O62 – Lys73^CO2^ interaction has lengthened (indicated by the gray line). In both parts of the MD trajectory, water molecules (red spheres) that enter the DWP remain >4 Å from the scissile bond (indicated by the red lines), and maintain hydrogen bonding contacts with the O62 atom. In both panels, the 6α-HE group is in a type-III rotamer.

**Table S1. Sequence and structure comparison of seven major *Acinetobacter* CHDL families***^a^*.

|  | OXA-23 | OXA-24/40 | OXA-51 | OXA-58 | OXA-134*^b^* | OXA-143 | OXA-213*^b^* |
| --- | --- | --- | --- | --- | --- | --- | --- |
| OXA-23 | – | 60 | 58 | 48 | 57 | 61 | 58 |
| OXA-24/40 | 0.57  235 | – | 62 | 48 | 57 | 61 | 58 |
| OXA-51 | 0.61  238 | 0.64  241 | – | 50 | 56 | 63 | 78 |
| OXA-58 | 1.02  236 | 1.11  239 | 1.09  238 | – | 51 | 51 | 47 |
| OXA-134 | 0.44  237 | 0.51  241 | 0.60  241 | 1.12  241 | – | 57 | 58 |
| OXA-143 | 0.76  237 | 0.75  241 | 0.76  241 | 1.11  238 | 0.76  240 | – | 64 |
| OXA-213 | 0.62  235 | 0.57  241 | 0.41  239 | 1.08  238 | 0.54  240 | 0.73  240 | – |

*^a^* The upper sector gives the pairwise sequence identity as a percentage. The lower sector gives the pairwise superposition *rmsd* (Å) and the number of Cα atoms matched.

*^b^* There are no structural representatives of the OXA-134 and OXA-213 families, so AlphaFold2 models (1) were used for the structural superpositions.

**Table S2. Data collection and refinement statistics for apo-OXA-23 and the**

**OXA-23-meropenem complex*^a^*.**

|  | apo-OXA-23 | meropenem |
| --- | --- | --- |
| *Data Collection* |  |  |
| Unit cell, a, b, c (Å) | 82.77, 82.77, 84.89 | 82.99, 82.99, 85.13 |
| Space group | P4_2_2_1_2 | P4_2_2_1_2 |
| Resolution (Å) | 37.8-1.40 (1.42-1.40) | 37.9-1.60 (1.63-1.60) |
| Reflections - observed  - unique | 1516028  58626 | 497837  39875 |
| *R*_meas_*^b^* | 0.080 (2.492) | 0.066 (1.010) |
| *R*_pim_*^c^* | 0.016 (0.505) | 0.018 (0.385) |
| *I* / σ*I* | 22.9 (1.3) | 20.7 (1.7) |
| Completeness (%) | 100 (100) | 100 (99.4) |
| CC½*^d^* | 1.0 (0.744) | 0.999 (0.625) |
| Average multiplicity | 25.9 (23.8) | 12.5 (6.5) |
| Wilson B (Å^2^) | 20.7 | 26.5 |
| *Refinement* |  |  |
| PDB Code | 9NSW | 9NT0 |
| *R*_work_ / *R*_free_*^e^* | 0.1662 / 0.2007 | 0.1742 / 0.2052 |
| Reflections, work/free | 58555 / 2926 | 39822 / 1949 |
| Number of atoms - protein  - water  - ligands | 2048  185  - | 2011  135  26 |
| B-factors (Å^2^) - protein  - water  - ligands | 30.6  45.6  - | 28.2  35.5  41.0 |
| *rmsd*s - bond lengths (Å)  - bond angles (º) | 0.005  0.81 | 0.006  0.98 |
| Ramachandran plot*^f^* - favored (%)  - outliers | 98.4  0 | 98.3  0 |
| Molprobity Score*^f^* | 0.92 (100^th^ percentile) | 1.15 (99^th^ percentile) |
| Molprobity Clashscore*^f^* | 1.69 (99^th^ percentile) | 1.95 (99^th^ percentile) |

*^a^* Numbers in parentheses refer to the highest resolution shell.

*^b^* *R*_meas_ is the redundancy-independent merging R factor (2).

*^c^* *R*_pim_ is the precision-indicating merging R factor (2).

*^d^* Correlation between intensities from random half-sets of data (3).

*^e^* *R*_free_ was calculated using a test set comprising 5% of the data randomly chosen.

*^f^* Calculated with the program MOLPROBITY (4).

**Table S3. Data collection and refinement statistics for the OXA-23-NA-1-157 complexes*^a^*.**

|  | 3 min | 4 min | 6 min |
| --- | --- | --- | --- |
| *Data Collection* |  |  |  |
| Unit cell, a, b, c (Å) | 82.98, 82.98, 84.98 | 82.47, 82.47, 84.43 | 82.77, 82.77, 86.04 |
| Space group | P4_2_2_1_2 | P4_2_2_1_2 | P4_2_2_1_2 |
| Resolution (Å) | 37.8-1.57 (1.60-1.57) | 37.6-1.70 (1.73-1.70) | 38.2-1.80 (1.84-1.80) |
| Reflections - observed  - unique | 545977  42061 | 417231  32604 | 366761  28335 |
| *R*_meas_*^b^* | 0.065 (1.294) | 0.095 (1.477) | 0.101 (1.283) |
| *R*_pim_*^c^* | 0.018 (0.371) | 0.026 (0.407) | 0.028 (0.369) |
| *I* / σ*I* | 21.4 (1.8) | 15.4 (1.5) | 16.1 (1.7) |
| Completeness (%) | 99.9 (99.0) | 99.8 (99.2) | 99.9 (98.3) |
| CC½*^d^* | 1.0 (0.810) | 0.999 (0.814) | 0.999 (0.807) |
| Average multiplicity | 13.0 (11.7) | 12.8 (12.6) | 12.9 (11.7) |
| Wilson B (Å^2^) | 22.5 | 25.2 | 27.9 |
| *Refinement* |  |  |  |
| PDB Code | 9NSX | 9NSY | 9NSZ |
| *R*_work_ / *R*_free_*^e^* | 0.1801 / 0.2054 | 0.1963 / 0.2294 | 0.1726 / 0.1995 |
| Reflections, work/free | 42003 / 2065 | 32444 / 1642 | 28273 / 1389 |
| Number of atoms - protein  - water  - ligands | 1980  140  26 | 1983  98  26 | 1998  143  26 |
| B-factors (Å^2^) - protein  - water  - ligands | 26.2  32.9  30.4 | 31.3  35.0  35.5 | 30.5  38.4  37.8 |
| *rmsd*s - bond lengths (Å)  - bond angles (º) | 0.006  1.02 | 0.006  1.11 | 0.007  0.944 |
| Ramachandran plot*^f^* - favored (%)  - outliers | 98.3  0 | 98.8  0 | 98.8  0 |
| Molprobity Score*^f^* | 0.95 (100^th^ percentile) | 1.14 (99^th^ percentile) | 1.23 (99^th^ percentile) |
| Molprobity Clashscore*^f^* | 1.25 (99^th^ percentile) | 2.47 (99^th^ percentile) | 2.45 (99^th^ percentile) |

*^a^* Numbers in parentheses refer to the highest resolution shell.

*^b^* *R*_meas_ is the redundancy-independent merging R factor (2).

*^c^* *R*_pim_ is the precision-indicating merging R factor (2).

*^d^* Correlation between intensities from random half-sets of data (3).

*^e^* *R*_free_ was calculated using a test set comprising 5% of the data randomly chosen.

*^f^* Calculated with the program MOLPROBITY (4).

**REFERENCES**

1. Stasyuk A, Smith CA. 2025. Standardized residue numbering and secondary structure nomenclature in the class D β-lactamases. ACS Infect Dis 11:805-812. doi:10.1021/acsinfecdis.5c00060.

2. Weiss MS. 2001. Global indicators of X-ray data quality. J Appl Crystallogr 34:130-135. doi:10.1107/S0021889800018227.

3. Karplus PA, Diederichs K. 2012. Linking crystallographic model and data quality. Science 336:1030-1033. doi:10.1126/science.1218231.

4. Chen VB, Arendall WB, 3rd, Headd JJ, Keedy DA, Immormino RM, Kapral GJ, Murray LW, Richardson JS, Richardson DC. 2010. MolProbity: all-atom structure validation for macromolecular crystallography. Acta Crystallogr Sect D: Biol Crystallogr 66:12-21. doi:10.1107/S0907444909042073.
